# Nanomaterial-Enabled Fiber-Optic SPR Biosensor for Continuous and Noninvasive Body Fluid Monitoring:Progress and Prospects

**DOI:** 10.3390/nano16150936

**Published:** 2026-07-29

**Authors:** Wenhan Ma, Zhilai Zhang, Jiayang Wang, Yulin Zhang, Zhe Gao, Hongji Zhang, Runze Hou, Pengcheng Tao, Xinlei Zhou

**Affiliations:** 1 School of Optoelectronic Engineering and Instrumentation Science, Dalian University of Technology, Dalian 116024, China; manwenhan123@mail.dlut.edu.cn (W.M.); 2777352985@mail.dlut.edu.cn (Z.Z.); stephen1305670643@mail.dlut.edu.cn (J.W.); zhangyulin@mail.dlut.edu.cn (Y.Z.); 993870445@mail.dlut.edu.cn (Z.G.); zhanghongji@mail.dlut.edu.cn (H.Z.); 201992239@mail.dlut.edu.cn (R.H.); pctao@dlut.edu.cn (P.T.); 2 Central Hospital of Dalian University of Technology, Dalian 116021, China

**Keywords:** SPR, fiber-optic biosensor, nanomaterial, body fluid monitoring, noninvasive monitoring

## Abstract

Continuous and noninvasive body fluid monitoring has attracted increasing attention in personalized healthcare, chronic disease management, and wearable point-of-care testing. Fiber-optic surface plasmon resonance (SPR) biosensors are particularly promising for this purpose because they combine label-free and real-time with miniaturization and low sample volume requirements. However, current body fluid sensing technologies and conventional bare metal SPR interfaces still face critical challenges, including insufficient analytical accuracy in complex biofluids, broad resonance linewidths, weak signal readability for trace biomarkers, and mechanical perturbations during wearable operation. These limitations highlight the need for nanomaterial-engineered fiber-optic SPR platforms that can convert interfacial molecular events into stable and sensitive signals. The review summarizes recent progress in nanomaterial-enabled fiber-optic SPR biosensors for continuous body fluid monitoring. Emphasis is first placed on nanomaterial mediated local electromagnetic field enhancement and plasmonic mode regulation. Subsequent discussion focuses on their functions in interfacial recognition, analyte enrichment, rapid mass transport, antifouling protection, and flexible integration for continuous operation. On this basis, representative sensing targets, material strategies, and device architectures for tears, urine, exhaled breath condensate, saliva and sweat are systematically analyzed. Finally, current challenges and future opportunities are discussed from the perspective of sensing reliability, wearable integration, and real sample validation.

## 1. Introduction

Continuous, noninvasive, and molecularly resolved health monitoring is increasingly recognized as a strategic direction for precision medicine, chronic disease management, and telehealth [1,2,3]. In contrast to conventional blood testing depending on invasive sampling and discrete measurement points, sensing approaches based on body fluids such as sweat [4], saliva [5], tears [6], urine [7], exhaled breath condensate [8], and interstitial fluid [9] can acquire continuous time-series information with a substantially lower physiological burden. Such measurements are better suited to capturing real-time metabolic fluctuations, inflammatory dynamics, drug response, and disease progression [10,11,12]. Recent advances in wearable body fluid analysis have already demonstrated the feasibility of generating clinically interpretable and remotely accessible biochemical data streams, even though analytical accuracy, long-term stability, and translational deployment remain major unresolved challenges [1,3].

Among the available sensing technologies, surface plasmon resonance (SPR) has long been regarded as a powerful platform for probing biomolecular interactions and performing optical bioanalysis because it is label-free, real-time, interfacially sensitive, and compatible with kinetic analysis [13,14,15,16]. SPR enables highly sensitive in situ detection of molecular binding events and minute refractive index (RI) change while providing access to surface coverage, adlayer thickness, binding kinetics, and affinity constants [17,18]. At the same time, reproducibility, compatibility with complex matrices, and robust data interpretation continue to limit its broader deployment in realistic bioanalytical settings [13].

Compared with conventional prism-based SPR, fiber-optic SPR retains the strong interfacial sensitivity of propagating plasmonic sensing while offering additional advantages, including miniaturization, remote signal transmission, structural flexibility, and compatibility with low sample volumes [2,19]. These characteristics make fiber-optic SPR particularly suitable for coupling with wearable platforms, microfluidic sampling strategies, and portable readout systems [20]. Nevertheless, when the plasmonic interface relies only on a bare Au or Ag film, the system often falls short of the requirements of continuous body fluid monitoring. The limited penetration depth of conventional propagating SPR weakens sensitivity to large biomolecular targets, multilayer adsorption, and recognition events relocated away from the plasmonic surface after flexible encapsulation [13]. In addition, broad resonance linewidths, imperfect field confinement, and low signal-to-noise ratios compromise trace analysis, long-term peak tracking, and drift correction. Bare metal surfaces are also vulnerable to nonspecific adsorption, oxidation, and mechanical perturbation in complex biofluids, which degrades selectivity, reproducibility, and steady-state readout performance [21].

Against this background, nanomaterial-enabled functional enhancement has become a decisive route for advancing fiber-optic SPR toward continuous body fluid monitoring. Recent reviews have emphasized that SPR biosensing is undergoing a transition from homogeneous thin film platforms to precisely engineered nanostructured interfaces [13,14,15,19,21]. Metallic nanoparticles, two-dimensional materials, meta surfaces, and composite heterostructures can substantially improve sensing performance through local field enhancement, interfacial charge transfer, exciton–plasmon coupling, and resonance-shape engineering. For fiber-optic SPR, the role of nanomaterials has therefore expanded far beyond simple RI sensitivity enhancement to mode engineering [14,22,23], interfacial recognition [24,25,26], analyte enrichment and mass transfer control [27,28,29], antifouling, and flexible integration [30,31,32]. However, the reviews do not systematically use the requirements of continuous and noninvasive body fluid monitoring as the central framework for connecting nanomaterial function, fiber-optic SPR performance, and device-level deployment.

**Figure 1:**
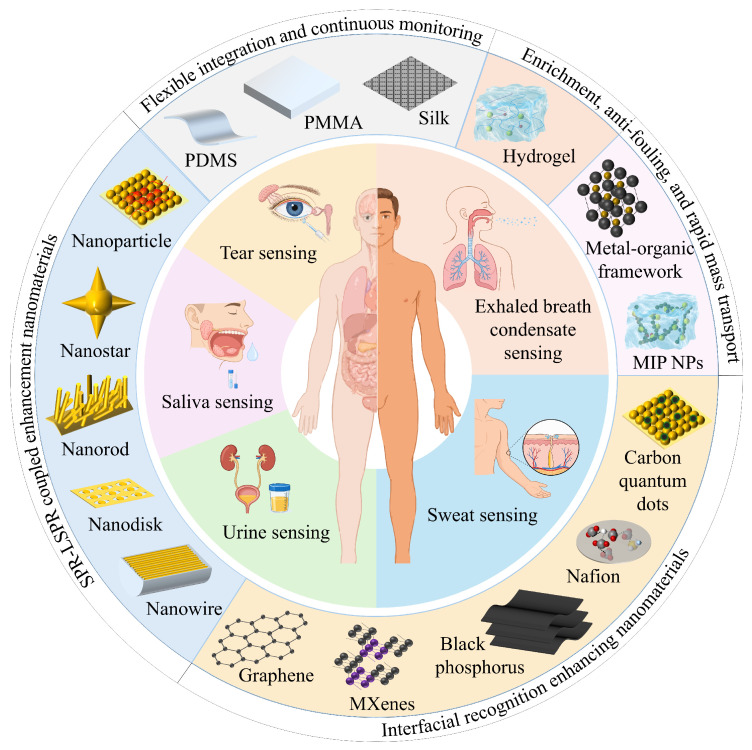
Schematic overview of nanomaterial-enabled fiber-optic SPR biosensing strategies for continuous and noninvasive body fluid monitoring. The central panel illustrates representative biofluid sensing scenarios, including tears, saliva, urine, exhaled breath condensate, and sweat analysis. The outer annulus summarizes the major classes of functional nanomaterials and soft interfaces used to advance fiber-optic SPR biosensors toward wearable and continuous monitoring platforms.

The present review is organized from the perspective of the end-use scenario of continuous and noninvasive body fluid monitoring. Beyond conventional figures of merit such as sensitivity and limit of detection, particular emphasis is placed on deployment-relevant parameters, including the full width half maximum (FWHM), figure of merit (FOM), long-term drift, antifouling capability, adaptability to flexible packaging, and clinical interpretability. Section 2 introduces SPR physics, interrogation methods, and performance metrics. Section 3 classifies nanomaterials, as shown in Figure 1, according to their roles in mode enhancement, interfacial recognition, enrichment, antifouling and mass transport, and flexible integration, highlighting how material functions govern device performance and application suitability. Section 4 discusses the key bottlenecks, optimal nanomaterial selection, and promising device architectures for tears, urine, exhaled breath condensate, saliva, and sweat. Section 5 summarizes the main conclusions and the challenges in translating proof-of-concept systems into clinically and domestically deployable technologies.

## 2. SPR Sensing

### 2.1. Principles of SPR and Related Modalities

SPR originates from momentum matched coupling between incident electromagnetic radiation and the collective oscillation of free electrons at a metal–dielectric interface. In electromagnetic terms, SPR occurs at an interface between a metal with a negative real dielectric constant and a dielectric with a positive real dielectric constant. A plasmon is a collective oscillation of free conduction electrons relative to the positive ionic background. At a metal–dielectric interface, coupling of this charge oscillation with the electromagnetic field forms a surface plasmon polariton (SPP). For a planar interface, the propagation constant of the SPP can be expressed as [33]

(1) $$k_{SPP}=\frac{\omega }{c}\sqrt{\frac{\varepsilon_{m}\varepsilon_{d}}{\varepsilon_{m}+\varepsilon_{d}}},$$

where ω is the angular frequency of the incident field, *c* is the speed of light, and $\varepsilon_{m}$ and $\varepsilon_{d}$ are the dielectric constants of the metal and dielectric medium, respectively.

**Figure 2:**
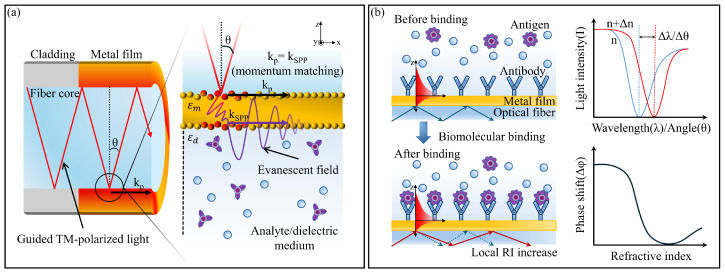
(**a**) Principle of fiber-optic SPR biosensor. (**b**) Schematic diagram of the interactions between the SPR fiber-optic biosensor and analyte.

Because the in-plane momentum of light in free space is insufficient to excite this surface mode directly, additional momentum must be supplied by a prism, a grating, a waveguide, or the evanescent field generated in a fiber-optic [34]. In fiber-optic SPR, as shown in Figure 2a, guided transverse magnetic (TM)-polarized light undergoes total internal reflection and generates an evanescent field at the unclad, metal-coated fiber surface. The parallel wave-number component of p-polarized light can be written as

(2) $$k_{p}=\frac{\omega }{c}\sqrt{\varepsilon_{p}}\sin\theta ,$$

where $\varepsilon_{p}$ is the dielectric constant of the coupling medium and θ is the incidence angle.

After a part of beam in core coupled into fiber cladding, the evanescent field generated excites the surface plasmons (SPs). When $k_{p}$ matches $k_{SPP}$, optical energy is efficiently transferred into the interfacial plasmon mode, giving rise to a pronounced resonance dip in the reflected or transmitted spectrum. This resonance condition underpins the sensing function of SPR. In Figure 2b, target binding increases the effective RI and interfacial mass loading within the evanescent-field region. This perturbation shifts the resonance wavelength or angle and changes the phase/intensity response, providing the measurable biosensing signal.

Around the resonance angle, the SPPs propagate along the metal film interface as strong evanescent waves, with oscillatory attenuation and exponential decay normal to the surface. The resulting field enhancement underpins the exceptional sensitivity of SPR to changes in the surrounding dielectric medium. Meanwhile, the evanescent-field penetration depth and the surface plasmon propagation length jointly define the effective sensing volume of SPR [13]. In addition, the change in RI is proportional to the mass of biomolecules bound to the metal surface [35]. In this sense, SPR output is an optical projection of interfacial mass loading and local electromagnetic redistribution. This combination of interfacial specificity, kinetic readability, and high sensitivity explains the enduring relevance of SPR in bioanalysis, drug screening, and continuous health monitoring.

Beyond conventional propagating SPR, a series of derivative modes have emerged to address different sensing demands. Localized SPR (LSPR) is the most common nanomaterial mediated enhancement mechanism in fiber sensors. Resonant excitation of subwavelength metallic nanostructures generates intense local fields near the particle surface, thereby improving sensitivity to low-abundance analytes [36]. From the perspective of continuous body fluid monitoring, the modal variants are not merely theoretical refinements; they directly address application-dependent needs such as stronger field localization for low volume samples [37], deeper penetration for proteins or extracellular vesicles [38], or narrower linewidths for long-term drift resistant readout [39].

### 2.2. Performance Evaluation of SPR Sensors

SPR systems can be interrogated through angular [40], wavelength [41], intensity [42], phase [43], or spatial displacement modulation [44]. In angular interrogation, the incident wavelength is fixed while the incidence angle is scanned to obtain the SPR reflectance curve. A change in the RI of the analyte or sensing interface modifies the SPP phase-matching condition, thereby shifting the resonance angle θ_res_. Conventional prism-based systems predominantly use angular scanning, which is stable and well established but less suitable for rapid and portable operation because it depends on mechanical rotation.

Fiber-optic SPR platforms more commonly employ wavelength or intensity interrogation. Wavelength modulation employs a broadband light source while maintaining a constant incident angle, and the SPR response is determined by tracking shifts in the resonance wavelength. In amplitude modulation, both the incident angle and wavelength are kept fixed, and changes in the resonance are evaluated by measuring variations in the reflected or transmitted light intensity. Wavelength tracking generally provides better resistance to drift and higher quantitative consistency, whereas intensity-based readout is attractive for low cost and high-speed portable devices [36]. In continuous and noninvasive sensing scenarios, performance assessment should therefore extend beyond a single sensitivity value and instead compare sensing structures across a multidimensional set of metrics that reflect spectral quality, noise tolerance, and deployability.

All interrogation schemes quantify the same RI-induced displacement or distortion of the SPR resonance, but express the response in different measurement domains. Table 1 summarizes the most commonly used performance parameters for fiber-optic SPR systems, where $x_{\mathrm{res}}$ denotes the response of different interrogation signals to RI changes, corresponding to the resonance wavelength $\lambda_{res}$, the resonance angle $\theta_{res}$, or other measurable resonance parameters. In practice, no single metric is sufficient. A sensor with high bulk RI sensitivity may still be unsuitable for longitudinal body fluid monitoring if the resonance feature is excessively broad, the figure of merit is low, or the interface drifts under prolonged exposure to complex biofluids [45,46,47,48]. Metrics such as FWHM, FOM, detection accuracy, long-term stability, and tolerance to fouling and mechanical perturbation are therefore essential for evaluating whether a given SPR architecture is genuinely relevant for practical continuous monitoring [49,50,51].

## 3. Functional Roles of Nanomaterials in Health Monitoring

For conventional Au or Ag film SPR sensors, bottlenecks often emerge in linewidth, stability, resistance to interference, and the detection of ultra-low abundance analytes [45,52]. More fundamentally, the core challenges faced by fiber-optic SPR in health monitoring expose three intrinsic limitations of classical propagating modes, namely limited field penetration depth [53,54], broad resonance features that compromise tracking precision [46], and insufficient interfacial functionality in complex biological media [50,55]. These limitations become more severe under wearable operating conditions, where samples are small, ionic strength and pH vary significantly, background molecules are abundant, and the sensor must remain flexible, low power, and mechanically stable during daily activity.

For this reason, recent advances in SPR are not defined simply by reiterations of the Kretschmann geometry, but by the emergence of mode engineering and nanomaterial based functional enhancement. In the context of continuous body fluid monitoring, nanomaterials can be grouped according to the role they play in the overall sensing system. Those are mode-enhancing materials, interfacial recognition-enhancing materials, enrichment/anti fouling/rapid mass transport materials, and materials that enable flexible integration and long-term wearable operation.

### 3.1. Mode-Enhancing Nanomaterials

Mode-enhancing nanomaterials directly reshape the electromagnetic field distribution, coupling strength, radiative loss, or resonance linewidth of hybrid SPP/LSPR states. When the dielectric constant changes from ε to ε+δε, the shift in the wavevector δk is proportional to the overlap integral:

(3) $$\delta k\propto \frac{\int_{V_{\mathrm{int}}}{\vec{E}}_{i}^{\;*}\;\delta \varepsilon \;{\vec{E}}_{f}\;dr}{\int_{V}{\vec{E}}_{i}^{\;*}\;\varepsilon \;{\vec{E}}_{i}\;dr},$$

where $E_{i}$ is the electrical field before the variation in the analyte RI took place, $E_{f}$ is the field after the index perturbation, and $V_{int}$ is the interaction volume.

At the electromagnetic level, nanomaterial overlayers reshape the dielectric environment and boundary conditions governing SPP propagation, thereby modifying phase matching and redistributing the modal energy between the sensing medium and the layers. Their primary task is to increase the field overlap within the analyte region and strengthen plasmonic interactions so that RI perturbations or interfacial binding events can be translated into larger and more readable optical signals. However, an effective enhancement layer must achieve increased field confinement within the biofluid while avoiding excessive absorption losses, scattering, and radiative damping. Importantly, the frontier of mode enhancement has shifted away from maximizing sensitivity alone. Current designs aim instead for coordinated optimization of sensitivity, spectral sharpness, stability, and practical suitability for continuous monitoring [19]. Based on the above requirements, the recent development of mode-enhancing nanomaterials is summarized from three perspectives.

#### 3.1.1. Metal Film SPR-LSPR Coupled Structures

**Figure 3:**
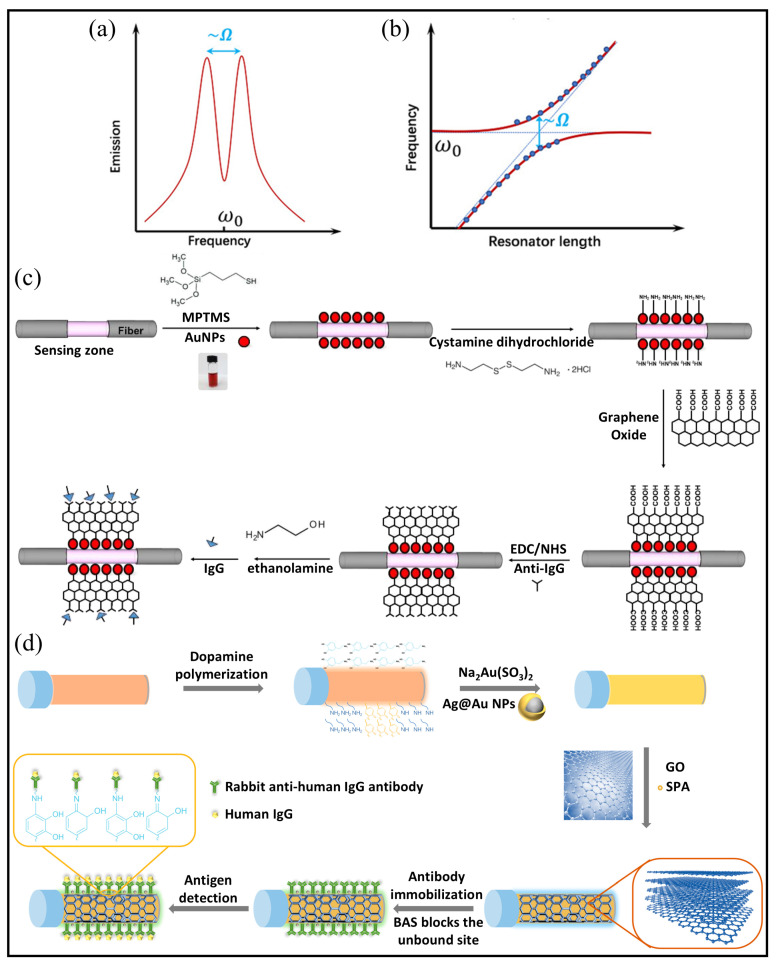
Metal film SPR-LSPR coupling and biofunctionalized nanointerfaces for enhanced fiber-optic sensing. (**a**) Splitting of the resonances around the resonance frequency of the exciton $\omega_{0}$; (**b**) anti-crossing behavior of the resonances when the cavity resonance is detuned around the exciton resonance [58]. (**c**) Schematic of the steps involved in the fabrication of gold nanoparticle/graphene oxide (AuNP/GO) sensor fiber [60]. (**d**) Schematic diagram of the preparation of graphene oxide-enhanced silver-nucleated gold-shell bimetallic fiber SPR sensor and antigen–antibody immunodetection [61]. (Reprinted with permission, copyright of WILEY and MDPI).

Coupling between propagating SPR in a metal film and LSPR in adjacent metallic nanoparticles is the earliest and most intuitive route for field enhancement [56]. When gold or silver nanoparticles (AuNPs or AgNPs) are placed near a plasmonic fiber surface, the localized resonance of the particle hybridizes with the propagating SPP supported by the film, generating a mixed mode within the nanoparticle (NP)-film-dielectric nanocavity. This hybrid state typically produces higher local field intensities and larger surface mass loading responses than the bare film alone, introducing a localized resonance channel into the original dispersion relation [57]. In the strong coupling limit, mode splitting and anti-crossing can occur, enabling dual-peak or ratio-based readout strategies that improve both sensitivity and precision [58,59], as shown in Figure 3a,b. From the broader perspective of plasmon hybridization, nanoparticle substrate systems can outperform simple dimers because substrate induced image charges and coupling to extended SPP states create richer hybrid mode landscapes [14].

Spherical AuNPs and AgNPs remain widely used because they are easy to synthesize, thiolate, bioconjugate, and secondary enhance of SPR [62,63]. Parts of fiber-optic platforms have used AuNP-enhanced surfaces to amplify immunorecognition and translate local field enhancement into real analytical gains. For example, Chen et al. introduced a AuNP/graphene oxide (GO) composite into a fiber-optic plasmon resonance platform for IgG detection in 2021, in which AuNPs functioned both as LSPR amplification units and as mass-amplifying labels for biomolecular recognition (Figure 3c) [60]. More recently, a silver-nucleated gold-shell bimetallic nanoparticles combined with GO was proposed to further enhance fiber SPR biosensing [61], where the Ag core strengthened the plasmonic response, the Au shell ensured chemical stability and biofunctionalization capability and GO improved interfacial coupling while offering abundant binding sites for protein immobilization [64]. The fabrication process is schematically illustrated in Figure 3d. The resulting device achieved an RI sensitivity of 4715.9 nm/RIU, and an IgG sensitivity of 0.53 nm/(μg/mL), demonstrating that rational nanostructure engineering can be directly translated into measurable gains in biological detection performance.

The practical significance of these coupled structures is especially clear in continuous body fluid sensing, where the target is often not a bulk RI change but a small number of interfacial binding events occurring in a low volume and compositionally complex sample [65]. In addition, beyond serving as a model analyte, antigen-specific IgG in saliva has been used to assess infection history and immune exposure [66], while tear IgG has been explored in ocular biomarker studies [67]. In urine, elevated IgG excretion may reflect increased glomerular permeability and renal barrier injury [68]. Thus, IgG itself can carry clinically useful information in selected body fluids, but its translation to wearable continuous sensing requires careful control of biofluid matrix effects and long-term baseline stability.

#### 3.1.2. Anisotropic Nanostructures

Although spherical nanoparticles can provide LSPR enhancement, their resonance tunability, spectral sharpness, and hotspot intensity are limited [69]. These shortcomings are particularly problematic on fiber platforms, where curved incidence, multimode propagation, and nonuniform coatings tend to broaden resonance features [70,71,72]. So, it remains challenging to obtain an ideal high-Q resonance by relying solely on a continuous metal film [73]. By contrast, anisotropic nanostructures such as nanorods (NRs), nanostars (NSs), nanodisks (NDs), and nanowires (NWs) can dramatically enhance local field confinement through tip effects and longitudinal/transverse mode splitting, while also shifting the resonance window toward the near-infrared region that is often preferred for biosensing [74,75].

**Figure 4:**
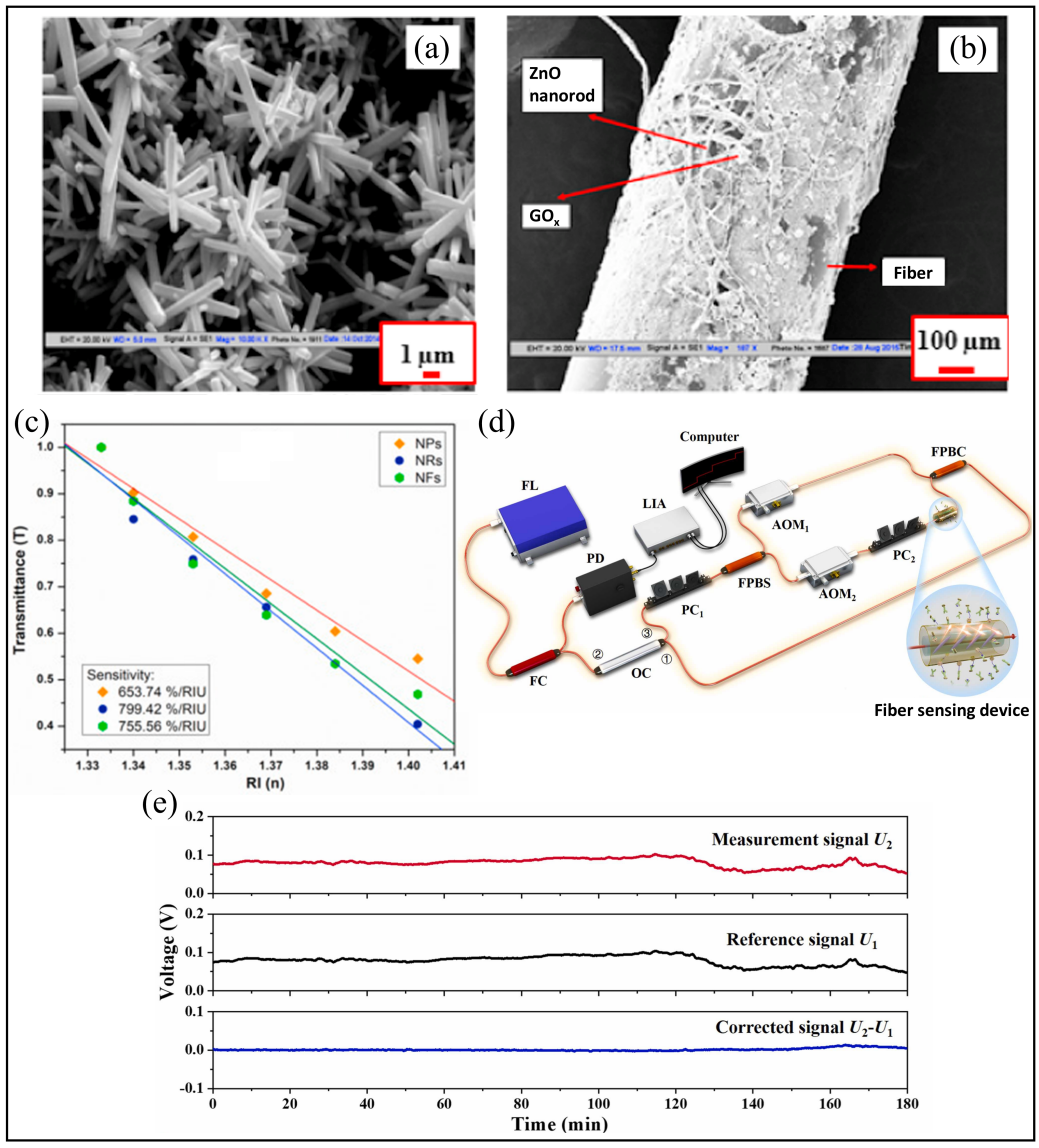
Anisotropic nanostructure-enabled field confinement and modal coupling in fiber-optic SPR sensors. (**a**) SEM images of the ZnO NRs; (**b**) SEM image of glucose oxidase (GO_x_) enzyme immobilized on ZnO NRs grown on optical fiber having silver layer coating [76] (**c**). Sensitivity variation of the sensor coated with ZnO NPs, NFs, and NRs [78]. (**d**) The schematic diagram of the optical fiber SPR biosensor. FL: fiber laser; FC: fiber coupler; PD: photodetector; LIA: lock-in amplifier; OC: optical circulator; PC1, PC2: polarization controller; FPBS: fiber polarization beam splitter; AOM1, AOM2: acousto-optic modulators; FPBC: fiber polarization beam combiner; (**e**) the 3 h test results of the RI sensor responding to water via the frequency multiplexing compensation method [79]. (Reprinted with permission, copyright of Elsevier).

Early evidence for the value of anisotropy came from rod-/wire-like high-aspect-ratio structures coupled to metallic fiber films. ZnO NRs, for example, as shown in Figure 4a,b, have been shown not only to provide a high surface area scaffold for enzyme immobilization, but also to extend the evanescent field into the surrounding sample because of their high RI and anisotropic morphology [76]. Kadhim et al. subsequently proposed a multiple D-shaped AuNWs/Fe_3_O_4_ SPR sensor, in which the elongated nanowire stimulated the plasmon mode concurrently strengthening guided-mode/plasmon coupling and improving resonance characteristics [77]. As shown in Figure 4c, systematically comparing ZnO NPs, nanoflowers (NFs), and vertically aligned NRs on the outer surface of no-core fibers, it can be found that ZnO NRs delivered the highest and most stable average sensitivity, reaching 799.42%/RIU over the RI range of 1.33–1.40 [78]. This advantage was attributed to the larger and more uniform effective surface area, better crystallinity, and more favorable dispersion characteristics associated with NR morphology. Taken together, these studies indicate that when nanostructures evolve from isotropic particles to highly oriented one-dimensional architectures, mode enhancement arises increasingly from the cooperative reconstruction of local field confinement and guided-mode leakage pathways.

However, in realistic body fluid measurements, continuous monitoring is inevitably affected by matrix fluctuation, temperature variation, and laser-intensity drift. These noise sources can obscure weak RI or molecular-binding signals even when the plasmonic field is strongly enhanced. In this regard, as shown in Figure 4d, Dai et al. integrated AuNRs into a fiber-optic SPR platform, combined with laser heterodyne feedback and frequency multiplexing compensation, achieving the RI resolution to $1.06\times 10^{-6}$ RIU [79]. As shown in Figure 4e, the incorporated readout-stabilization strategy effectively suppresses source-related intensity noise, compensates environmental disturbance, and improves long-term baseline stability. This development indicates that the role of anisotropic nanostructures has extended beyond peak shift amplification toward weak signal enhancement and long-term readout stabilization.

**Figure 5:**
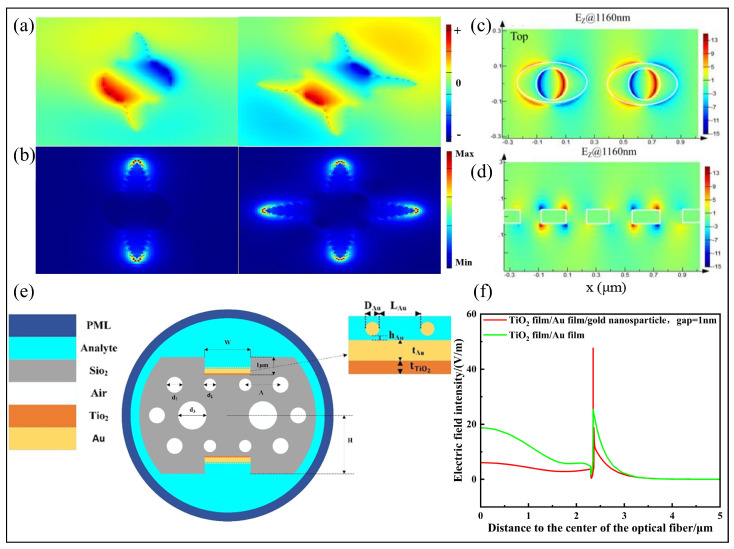
Effect of the number of the tips on the SPR of the gold nanostars. (**a**) Surface charge distribution and (**b**) electric field distribution excited at SPR of nanostars containing two and four protruding tips [23]. The z component of electric field E_z_ distributions located at different sections in the CNENA system: (**c**) at 2 nm distance from the top surface of gold film for 1160 nm, (**d**) through the center of binary structure along x–z plane for 1160 nm [80] (**e**). Cross-sectional view of D-shaped dual-sided slotted photonic crystal fiber surface plasmon resonance sensor; (**f**) when the analyte RI = 1.385 and surface plasmon resonance occurs simultaneously, the local electric field curve of TiO_2_ film/gold film and the local electric field curve of gold nanoparticles at a gap of 1 nm [81]. (Reprinted with permission, copyright of Elsevier, Springer Nature, and MDPI).

Subsequently, the emphasis of anisotropic structure research shifted toward morphologically tunable, high-Q coupled modes. As shown in Figure 5a,b, Chen showed for AuNSs that the dominant SPR response is governed primarily by tip length, with intense near fields localized around the tips and inter-tip gaps [23]. The principal SPR band can be tuned from the visible to the near-infrared region by increasing tip length, while exhibiting a high saturable absorption modulation depth up to 53%. Chen et al. advanced this concept from single particle LSPR enhancement to array enabled collective mode engineering by proposing a metallic circular ND-in-elliptical nanohole binary array (CNENBA) integrated on a fiber tip. In the near-infrared region, this design simultaneously achieved a sensitivity of 669 nm/RIU, a narrow linewidth of 5.8 nm, and a high figure of merit of 115.3, which the authors attributed to strong coupling between the nanodisks and elliptical nanohole array that suppressed radiative damping and promoted sharper plasmonic resonances [80] (Figure 5c,d).

By 2025, Khodatars Dashtmian et al. had further integrated AuNWs with a U-shaped open channel SPR-PCF architecture, combining anisotropic plasmonic elements with open microchannels, high throughput analyte injection, and near-infrared operation. Their design achieved a wavelength sensitivity of 7857.14 nm/RIU and an amplitude sensitivity of −1893.08 ${\mathrm{RIU}}^{-1}$ [82]. A 2026 study in Sensors reflects the current direction even more clearly. By combining an AuNP array with an Au/${\mathrm{TiO}}_{2}$ bilayer in a dual-channel D-shaped PCF (Figure 5e), the design exploited the synergistic coupling between propagating SPR and particle-assisted LSPR (Figure 5f), achieving an average wavelength sensitivity of 5733 nm/RIU. Although that work is primarily simulation-based, it captures the central trajectory of the field, namely, the pursuit of global coupling optimization among nanoparticle morphology, array spacing, dielectric thickness, and fiber architecture [81].

Anisotropic noble-metal structures offer simultaneous control over field intensity, resonance position, radiative loss, and linewidth. This makes them particularly relevant for continuous monitoring, where low background, narrow linewidth, minimal baseline drift, and long-term peak trackability are as important as absolute sensitivity. And now they are moving toward arrayed architecture, binary coupled structures, and high-Q mode engineering associated with Fano-like and quasi-bound states in the continuum (Quasi-BIC) responses [36,83]. Nevertheless, the performance of anisotropic structures is highly dependent on factors such as tip radius, orientation, and array order, which are difficult to reproduce uniformly on flexible or curved fiber surfaces. High-Q coupled modes may also become sensitive to fabrication defects and mechanical perturbations. Two-dimensional and high-index nanolayers therefore represent a logical next step, enabling thickness and dielectric controlled mode regulation together with subsequent biomolecular functionalization.

#### 3.1.3. Two-Dimensional and High Index Nanolayers

As mode engineering strategies have evolved, enhancement mechanisms have increasingly moved beyond noble-metal nanoparticles toward mixed architectures in which two-dimensional materials and high RI nanolayers actively participate in the plasmonic mode. These overlayers alter the effective dielectric environment, reshape evanescent field distribution, and tune the hybridization conditions between propagating and localized modes.

In many high-index oxides coated fiber-optic SPR configurations, ZnO, TiO_2_ or ITO modifies the effective modal index and the phase matching condition between the guided mode and the SPP, thereby regulating the resonance wavelength and interfacial field distribution, and often shifting the resonance toward longer wavelengths. Owing to their wide band gaps, ZnO and TiO_2_ generally exhibit relatively low optical absorption in the near-infrared spectral regions, allowing them to enhance the penetration ability of the evanescent field and thus increase the SPR intensity [84]. ITO is distinct from the wide-bandgap dielectric oxides because its complex permittivity is strongly dispersive and carrier-density-dependent; the plasmonic response of ITO can be tuned through carrier accumulation [85]. However, the resulting SPR spectral response will be affected by oxide thickness and layer sequence [86]. For multilayer nanostructured probes intended for continuous body fluid monitoring, these parameters should therefore be co-optimized to balance factors such as sensitivity, resonance linewidth, and fiber attenuation.

**Figure 6:**
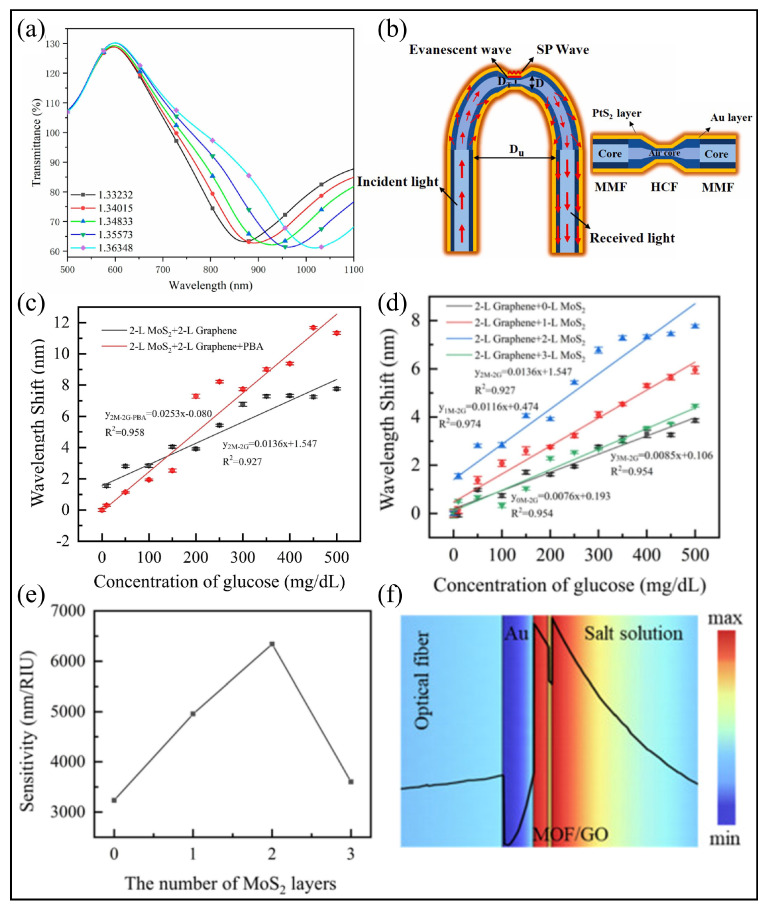
2D and high RI nanolayers for interfacial mode engineering and analyte-specific amplification in fiber-optic SPR sensors. (**a**) The RI experiment of the sensor by pulling the dispersion of InSe nanosheets at 40 times on the surface of the gold film. The Au film has a thickness of approximately 50–60 nm, while the InSe nanosheets have a thickness of around 40 nm [90]. (**b**) Schematic illustration of structure and working principle of U-tapered HCF SPR biosensor [91]. (**c**) Resonance wavelength shift versus glucose concentration for FO-SPR sensors with heterostructure and PBA; (**d**) resonance wavelength shift versus glucose concentration for FO-SPR sensors with different layers of MoS_2_; (**e**) the sensor sensitivity versus the number of MoS_2_ layers [92]. (**f**) Electric field profile for the MOF/GO-coated fiber-optic SPR sensors [93]. (Reprinted with permission, copyright of Elsevier).

Early work in this direction proposed a graphene/${\mathrm{MoS}}_{2}$/ZnO composite overlayer for enhancing fiber-optic SPR sensing [87]. Based on a multilayer Fresnel model, they showed that graphene can modulate the interfacial electromagnetic boundary and improve sensitivity, providing higher values of sensitivity. ${\mathrm{MoS}}_{2}$ can strengthen modal coupling through its strong optical absorption and increase the interaction of the bio-recognition molecules with the surface of the sensor [88,89]. ZnO, acting as a high RI layer, can further compress and extend the evanescent field into the analyte region. The ZnO/Ag configuration achieved a maximum theoretical sensitivity of 4740.9 nm/RIU. Nevertheless, this work remained purely theoretical and did not progress to real biological sample validation. Subsequently, Yin et al. later demonstrated experimentally that high mobility InSe nanosheets can actively reinforce local fields and plasmon propagation, achieving the sensitivity up to 4655.76 nm/RIU and the FOM value of 33.25 ${\mathrm{RIU}}^{-1}$ under the InSe thickness is 40 nm [90] (Figure 6a). Li and Wang further pushed this concept toward biosensing by combining a ${\mathrm{PtS}}_{2}$ overlayer with a U-tapered hollow-core fiber (Figure 6b), coupling structural field amplification with 2D-material-enabled interfacial enhancement for trace biomarker detection [91]. Collectively, these studies indicate that 2D-assisted fiber SPR has evolved from simple surface decoration to active mode engineering. Yet, many remain heavily simulation-dependent, most are validated primarily in RI liquids rather than real body fluids, and the added structural or multilayer complexity may compromise fabrication reproducibility, packaging, and long-term wearable compatibility.

The transition from individual 2D monolayers to heterostructure and multiphase functional layers became more explicit in 2025. A two-layer ${\mathrm{MoS}}_{2}$-graphene heterostructure fiber SPR glucose sensor was proposed for continuous glucose monitoring. Pyrene-1-boronic acid (PBA) was bound to the heterostructure to improve specificity for glucose molecules and sensitivity (up to 12,593.06 nm/RIU) [92] (Figure 6c). Notably, as detailed in Figure 6d,e, the work showed that increasing the number of 2D layers does not indefinitely improve sensor quality, because higher raw sensitivity can be offset by linewidth broadening and degraded spectral precision [94]. Such trade-offs are central to body fluid monitoring, where peak traceability, baseline stability, and noise tolerance are often more important than sensitivity alone.

A similar convergence of optical and biochemical functionality emerges in composite layers such as metal–organic frameworks (MOF)/GO [93] (Figure 6f), polyvinyl alcohol (PVA)/GO [95] and β-cyclodextrin (β-CD)/GO [96], which simultaneously tune the local dielectric environment, facilitate charge or field coupling, and provide chemically active, high surface area interfaces for analyte capture. However, these composite strategies still face substantial translation barriers, including uncertain long-term coating stability, incomplete antifouling validation, and limited evidence under mechanically perturbed, low-volume, real-sample conditions. Nanomaterials are beginning to transform fiber SPR from a RI transducer into a more integrated biosensing interface, but they have not yet fully solved the durability and robustness requirements imposed by longitudinal body fluid analysis.

Table 2 summarizes representative mode enhanced fiber-optic SPR sensors in terms of material systems, key performance metrics, and application scenarios. Metal film/LSPR coupled structures primarily amplify weak interfacial binding signals from low abundance biomarkers, but their practical use in biofluids still requires antifouling and baseline stabilizing interfaces. As material design advances toward anisotropic nanostructures, the emphasis shifts to regulating guided-mode/plasmon coupling, resonance position, and linewidth, which are critical for resolving small molecular-binding signals from matrix fluctuation and optical drift. However, long-term readout stability remains a key limitation. In contrast, 2D/high-index multilayers offer a more integrated strategy by combining field enhancement, molecular recognition, and analyte enrichment, but they also introduce inter-layer instability, resonance broadening, and insufficient validation in mechanically perturbed real biofluids.

### 3.2. Interfacial Recognition-Enhancing Nanomaterials

Unlike mode-enhancing materials, whose main role is to reshape the electromagnetic response, interfacial recognition-enhancing nanomaterials primarily reconfigure the molecular recognition and signal transduction processes at the metal/dielectric interface. Their importance is especially evident in continuous body fluid monitoring, where sweat, saliva, urine, and wound exudate represent chemically and biologically complex matrices characterized by high ionic strength, proteins and mucins, dynamic pH fluctuations, and persistent fouling at sensor interfaces [102,103,104]. Under such conditions, the true limitation is whether target molecules can be captured reproducibly, whether local charge perturbations can be efficiently translated into optical change, and whether baseline drift can be suppressed over long periods of operation [105].

Mechanistically, as listed in Table 3, this class of materials includes at least three subgroups. The first comprises two-dimensional materials such as graphene, GO, reduced GO (rGO), transition-metal dichalcogenides, MXenes, and BP, which enhance interfacial events through large specific surface area, abundant surface functionality, charge transfer, and exciton–plasmon coupling. The second includes high-index and semiconducting layers such as ZnO, TiO_2_, and ITO, which can both amplify RI perturbations and serve as scaffolds for receptor immobilization. The third comprises auxiliary interfacial materials such as CQDs, PDA, Nafion, MnO_2_, and stimuli-responsive hydrogels, which are useful for receptor loading, local isolation, anti-crosstalk operation, signal amplification, and long-term interfacial stabilization.

**Figure 7:**
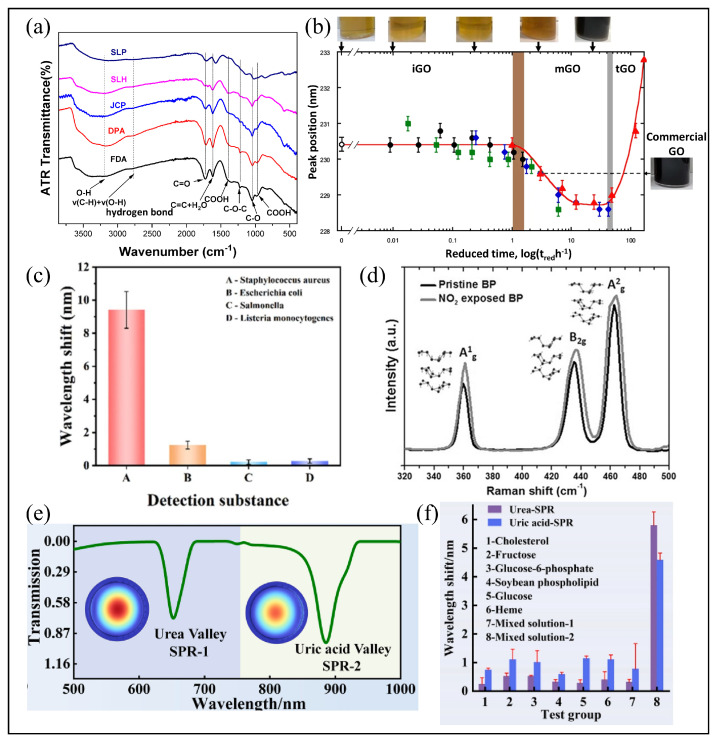
(**a**) ATR-FTIR spectra of commercial GOs [108]. (**b**) Reduced time course of the peak position of the π-π transition of GO colloids ripened at different temperatures for various durations. The reduced time course consists of three regions attributed to intrinsic GO (iGO), metastable GO (mGO) and transient GO (tGO). Brown and gray zones represent the transition regions. Ripening temperature: black circles, 298 K; green squares, 308 K; blue diamonds, 333 K; red triangles, 348 K [109]. (**c**) Comparison of wavelength shifts for the detection of various bacteria using a Ti_3_C_2_T_x_ MXene fiber SPR biosensor at equivalent concentrations [99]. (**d**) Raman spectra BP gas sensor before (black) and after (red) exposure to 100 ppm NO_2_ for 1 h showing qualitatively identical spectra of the pristine BP [112]. (**e**) SPR waveform output of optical fiber-gold film-ZnO- double enzyme model; (**f**) the wavelength value of the sensor when detecting interfering substances [113]. (Reprinted with permission, copyright of MDPI, Springer Nature, Elsevier, and WILEY).

The value of graphene-family materials lies in their ability to provide dense adsorption sites, rich surface chemistry, and partial protection of the underlying metal. Introducing graphene or GO into an SPR interface can improve loading efficiency and stability of antibodies, aptamers, or small molecule receptors attached on top [106]. However, as shown in Figure 7a, GO and rGO often exhibit variations in defect density as well as in the type and distribution of oxygen-containing functional groups, owing to differences in synthetic routes [107,108]. In addition, GO-based systems may undergo aging, swelling, and interfacial stability deterioration in aqueous environments [109,110] (Figure 7b). Therefore, for device design targeting continuous biofluid monitoring, graphene-family materials are more suitably integrated with PDA, molecularly imprinted layers, or oriented bioreceptors to construct composite recognition interfaces, achieving a better balance among sensitivity, specificity, and long-term operational stability [61,111].

More reactive materials such as TMDCs, MXenes, and BP offer a special combination of active interface and coupling enhancement. An fiber-optic SPR sensor enhanced by Ti_3_C_2_T_x_ MXene, for instance, demonstrated label-free bacterial detection with maximizing FOM of 17.88 ${\mathrm{RIU}}^{-1}$ and a low LOD of 1.14 CFU/mL when combined with PDA-mediated biofunctionalization [99]. High hydrophilicity, rich surface terminations, and the MXene multilayer’s strong absorption of evanescent field make it possible to read out binding events in complex samples with a higher sensitivity and a lower linewidth (Figure 7c). Compared with graphene-based counterparts, BP modified SPR structures were reported to exhibit about 1.4-fold higher sensitivity, owing to its puckered lattice, larger surface to volume ratio, and stronger molecular adsorption energy [112]. However, in hydrated and oxygen containing environments, as shown in Figure 7d, BP remains vulnerable to oxidation and performance drift.

Instead, high RI or semiconducting layers offer a more stable and engineerable mode of interfacial recognition enhancement. In 2025, Chen et al. proposed a fiber-optic dual-SPR biosensor assisted with ZnO-Nafion (Figure 7e), for simultaneous detection of urea and uric acid concentrations. ZnO nanoparticles are used as both the immobilization substrate for urease/uricase and the SPR sensitivity enhancement layer; a Nafion ion exchange membrane is introduced to coat the urea recognition area to block the extra sites and avoid crosstalk between adjacent detection areas. Figure 7f presents the sensor selectivity under different samples. Experiments indicate that the maximum sensitivity of urea and uric detection is 1.6 nm/mM and 36 nm/mM, respectively [113].

Similar arguments apply to highly editable functional materials (for example, CQDs, with high specific surface areas, a high density of organic functional groups, and good biocompatibility [25]), as well as to other auxiliary layers such as sensitive hydrogels that exhibit diverse swelling behaviors due to varying ionization degrees of carboxyl groups. High polarizability and dense surface functional groups of QDs increase the local RI perturbation and mass loading near the plasmonic interface; when the QDs excitonic emission band overlaps spectrally with the relevant SPP mode, the resonant coupling of QD’s spontaneous emission onto the propagating SPs may provide an additional signal-amplification pathway [114]. For longitudinal measurements in sweat, saliva, tears, or urine, however, the signal gain must be balanced against batch-to-batch variations in nanodot surface chemistry and composition-dependent toxicity [115].

### 3.3. Nanomaterials for Enrichment, Antifouling, and Rapid Mass Transport

**Figure 8:**
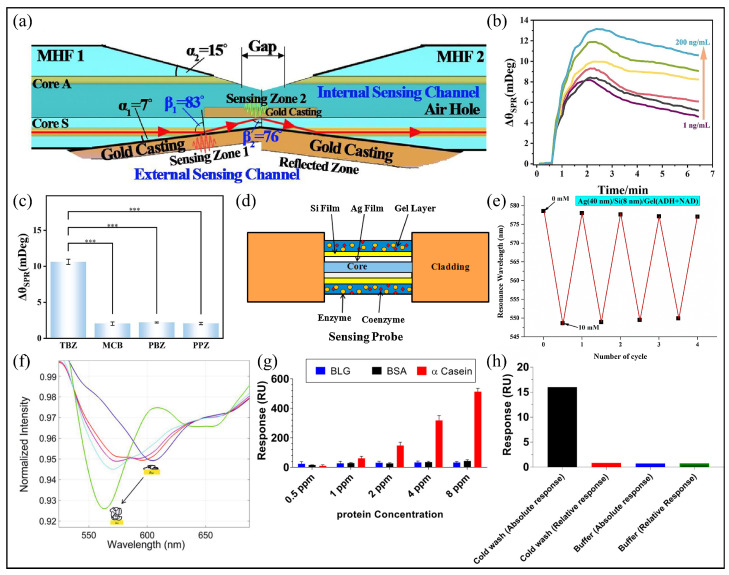
(**a**) Sketch diagram of the SPR sensing probe based on the MHF grinding-polishing butt-jointing technology [28]. Performance evaluation of tebuconazole (TBZ) detection: (**b**) sensor responses across varying TBZ concentrations; (**c**) specificity test results. *** indicates a statistically significant difference at p≤0.001 [116]. (**d**) Schematic diagram of ethanol sensing probe; (**e**) response of the sensor up to four cycles for the ethanol concentrations of 0 mM and 10 mM [117]. (**f**) The hydration process of nanoMIPs on the POF. Water was placed on the dry nanoMIP-POF, and the hydration process was optically followed over time. Spectra were collected at: t = 0 min, blue; t = 30 min, red; t = 40 min, magenta; t = 60 min, cyan; t = 180 min, green [118]. (**g**) Responses of α-casein, β-lactoglobulin, and bovine serum albumin BSA when injected at 0.5–8 ppm for a nanoMIP based sensor and (**h**) the relative baseline responses of the cold wash after gel filtration [119]. (Reprinted with permission, copyright of Elsevier and American Chemical Society).

After nanomaterials enable receptor immobilization and interfacial recognition, the central question of the realistic body fluid monitoring gradually shifts to how the sensing interface can simultaneously enrich analytes, resist contamination, and accelerate mass transport under low-volume and high-background conditions. An approach in this direction is the integration of fiber SPR with microfluidic sampling structures [28]. In Figure 8a, hollow and capillary fiber architectures have shown that internal and external sensing channels can be combined within a single fiber to reduce sample consumption and shorten the diffusion path between analytes and the plasmonic interface. These systems establish the structural basis for controlled fluid handling, but by themselves do not fully solve the problems of trace enrichment or antifouling. Those functions must be supplied by additional nanomaterial layers. Magnetic core-shell nanoparticles offer a more active approach. Fe_3_O_4_@Au@PDA nanoparticles, for example, can combine magnetic preconcentration, local RI perturbation, plasmonic signal amplification, and reduced nonspecific adsorption in a single unit [116]. The results of experiments are shown in Figure 8b,c. Their importance lies not only in stronger signal output but in the integration of enrichment, amplification, and interfacial protection into one controllable nano-object. But the additional actuation and regeneration procedures complicate uninterrupted wearable operation.

Metal–organic frameworks (MOFs) represent a second and increasingly important route. Because of their high specific surface areas, tunable porosity, and molecular sieving properties, MOFs can simultaneously promote analyte enrichment and efficient transport while acting as rigid interfacial scaffolds [120]. In recent years’ researches, ZIF-8/lipase composite layers [121], UIO-66-NH_2_-modified fiber SPR DNA sensors [122], and AgNPs@Cu-TCPP(Pt) (where Cu and Pt indicate copper and platinum, respectively, and TCPP(Pt) is meso-tetra (4carboxyphenyl) porphyrin)/Au/TFBG (tilted fiber Bragg grating) platforms for multi-ion detection [123] collectively show that MOFs have evolved from passive porous coatings into rigid multifunctional interfaces capable of sieving, enrichment, and anti-interference. Even so, their long-term use in biological fluids still faces challenges related to mechanical robustness, fouling accumulation, and wearable compatibility.

Hydrogel-based systems are closer to the practical demands of continuous body fluid sensing because their hydrated, permeable, and compliant networks more closely resemble physiological environments [124]. Hydrogel containing SPR interfaces can provide mild microenvironments for enzyme retention, tunable swelling assisted transport, and, in some cases, direct stimulus responsive transduction. As Zhao et al. proposed, a hydrogel was coated on the surface of the fiber-optic. The volume and RI of the gel change with the pH of the solution, resulting in the shift of the SPR wavelength, and a maximum pH sensitivity of 13 nm/pH was obtained [125]. In Semwal’s research, a probe coating films of silver and silicon was combined with hydrogel layer containing immobilized alcohol dehydrogenase (ADH) and its coenzyme (Figure 8d). The inorganic nanophase enhances local fields, whereas the hydrogel entrapped with enzyme and its coenzyme improves analyte transport and resistance to interfacial failure, obtaining the LOD of 15.34 μM and the LOQ of 29.63 μM [117]. Figure 8e demonstrates repeatable switching between 0 and 10 mM ethanol over four cycles, supporting the reversibility and short-term repeatability of the sensing interface.

Hydrogel molecularly imprinted polymer nanoparticles (MIP NPs) further extend this concept by coupling selective recognition with fast kinetics and reusable operation [29]. Compared with bulk or microscale imprinted materials, hydrogel MIP NPs provide more accessible binding sites, shorter diffusion distances, and lower nonspecific occupation, while preserving the hydrated and compliant character required for bio macromolecular recognition under mild aqueous conditions. A human transferrin (HTR) sensor was developed by Cennamo et al., in which soft MIP NPs underwent analyte induced deformation and amplified the plasmonic wavelength shift (Figure 8f), enabling a LOD of 1.2 fM over a 1.2 f–1.8 pM linear dynamic range [118]. The target binding in interface alters nanogel mechanics and strengthens the SPR response. The α-casein MIP NPs-SPR sensor reported by Ashley et al. highlights the resistance to matrix interference. Covalently immobilized MIP NPs enabled a LOD of 0.127 ppm over 0–150 ppm, together with satisfactory recoveries of 87–120% in α-casein spiked cleaning in place wash samples and clear selectivity over β-lactoglobulin and bovine serum albumin [119]. As shown in Figure 8g,h, although this is not yet equivalent to true long-term antifouling in physiological fluids, it does show that MIP NPs-functionalized soft interfaces can suppress nonspecific occupation more effectively than conventional bulk MIP layers.

Table 4 compares representative enrichment, antifouling, and rapid mass-transport schemes in terms of their key performance metrics, applications, and main limitations. These studies show that practical continuous biofluid sensing requires not only strong plasmonic enhancement, but also efficient analyte delivery, reduced nonspecific adsorption, and stable mass transport at the sensing interface.

### 3.4. Nanomaterials for Flexible Integration and Continuous Monitoring

**Figure 9:**
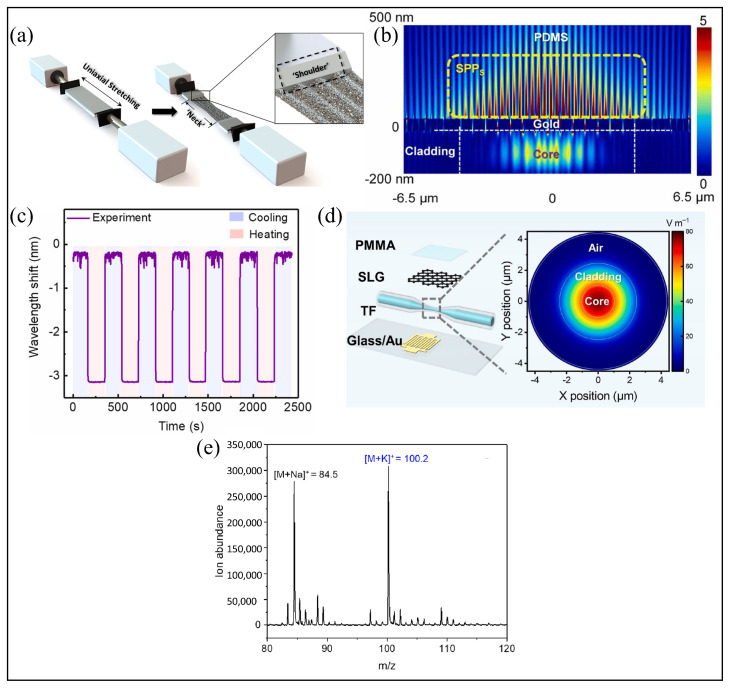
(**a**) The polymer SPR film is fixed onto a mechanical machine and the uniaxial stretching of polymer SPR film at a constant stretching speed [127]. (**b**) Electric-field intensity distribution of the plasmonic crystal structures coated with PDMS at 817 nm; (**c**) the real-time monitoring curve for a fiber-optic SPR device coated with PDMS hanging in air, as the heating stage was moved up and down cyclically [128]. (**d**) The simulation of on-fiber graphene/poly(methyl methacrylate) (PMMA) photodetector [129]. (**e**) LDI-MS spectrum of 100 mM urea in deionized water [32]. (Reprinted with permission, copyright of American Chemical Society and Elsevier).

To maintain high-performance interfaces under skin contact, bending, low-volume body fluid exposure, and long-term operation, flexible integration strategies embed enrichment, antifouling, and transport functions into wearable system architectures [2]. In this domain, hydrogels were among the first materials to move fiber SPR away from rigid metal-coated interfaces toward soft, body fluid compatible contact layers. Recent reviews on imprinted hydrogel nanoparticles suggest that these soft materials are evolving from passive support into multifunctional platforms that combine recognition, transport, antifouling, and wearable compliance [29]. The broader relevance of this evolution is underscored by flexible optical and spectroscopic sweat patches based on a sulfonated cellulose nanocomposite hydrogel (S-CNF-Ag NPs/PAA). Owing to its hydrophilic network, the hydrogel can absorb sweat, retain small molecules and enrich sweat analytes [126]. Figure 9a illustrates a flexible polymer SPR film subjected to controlled uniaxial stretching; the incorporation of cellulose and silver nanoparticles further imparts mechanical robustness, flexibility and strong interfacial adhesion, together with antimicrobial activity and good biocompatibility [127]. Such systems show that continuous noninvasive body fluid monitoring requires a soft material platform capable of sampling, conformal skin adhesion, antimicrobial protection, and stable signal transduction at the same time. The main limitations remain dehydration, swelling hysteresis, mechanical fatigue, and calibration drift during long-term wear.

Elastomeric materials such as PDMS enable the fragile fiber-optic probes to be incapsulated into a bendable, skin compatible and wearable structure. In the broader wearable fiber-optic literature, PDMS, polyethylene terephthalate (PET), and related polymers provide transparent encapsulation, mechanical buffering, and conformal contact [2]. Within plasmonic sensing specifically, thermosensitive PDMS coated fiber SPR microcavities developed by Kong et al. showed that flexible encapsulation can improve repeatability within ±0.3 °C, short-term response within 2 s, and low noise performance [128] (Figure 9b,c). It not merely softens the probe mechanically, but shifts optimization targets toward detection limits, repeatability, and response speed under realistic operating conditions. However, such encapsulation does not independently provide molecular recognition and may increase the distance between the recognition event and the region of strongest SPR near-field intensity.

The next stage of flexible integration concerns the readout mechanism itself. Traditional fiber SPR systems still depend heavily on spectrometers, which limit miniaturization and continuous wearability. On-fiber graphene/PMMA photodetectors have therefore been proposed to convert SPR signals directly into electrical output on the same fiber (Figure 9d). The SPR sensor demonstrated a detection sensitivity of 0.242 nm/(mg/mL) for urea and 1.059 nm/(mg/mL) for glucose, pointing toward compact, low-power, and electronically integrated wearable systems [129]. In parallel, capillary and hollow-fiber microfluidic SPR chips compress sampling and sensing into a single fiber-scale architecture for arctigenin detection. This small-sized and highly flexible all-fiber chip achieved the detection sensitivity of 0.205 nm/(μg/mL) and the LOD of 0.49 μg/mL, providing a solution for the high-density integrated packaging of flexible devices [30].

Table 5 summarizes the flexible-integration and continuous-monitoring schemes discussed above, including key metrics, applications, and limitations. Meanwhile, plastic optical fibers (POFs), photonic crystal fiber (PCF), silk substrates, and other soft platforms broaden the range of wearable carriers [130,131]. Promphet et al. fabricated flexible conductive silk patches by in situ AuNPs deposition followed by carbonization, enabling sweat urea detection at a 60 mM cut-off [32]. As shown in Figure 9e, the representative LDI-MS spectrum of 100 mM urea in deionized water demonstrates direct detection in positive-ion mode, confirming the carbonized AuNP–silk as an effective photoabsorbing and laser energy transfer substrate. POF has a low elastic modulus, a large core, and straightforward D-shaped or etched fabrication, facilitating conformal packaging, and low-cost coupling. A receptor-functionalized plasmonic POF detected cortisol in artificial saliva with a 96 fM LOD and an approximately 5 min response [24], while the Au/ITO POF study demonstrated good aqueous stability, showing only 1.2 nm of maximum resonance drift during an 11-day repeated-immersion test, and quantified the sensitivity–linewidth trade-off [132]. PCF, by contrast, offers microstructured control of effective index, birefringence, confinement loss, and analyte channels, enabling engineered phase matching and multi-parameter compensation. A 2025 three-channel Ag/MoS_2_ PCF platform covered with layers of hydrogel and PDMS experimentally combined cDNA concentration, pH, and temperature readout [133]. Because many published PCF architectures remain simulation-dominant, future continuous body fluid systems should prioritize capillary filling, fouling control, reference-channel isolation, and flexible connectorization rather than simulated peak sensitivity alone.

## 4. Future Prospects

**Figure 10:**
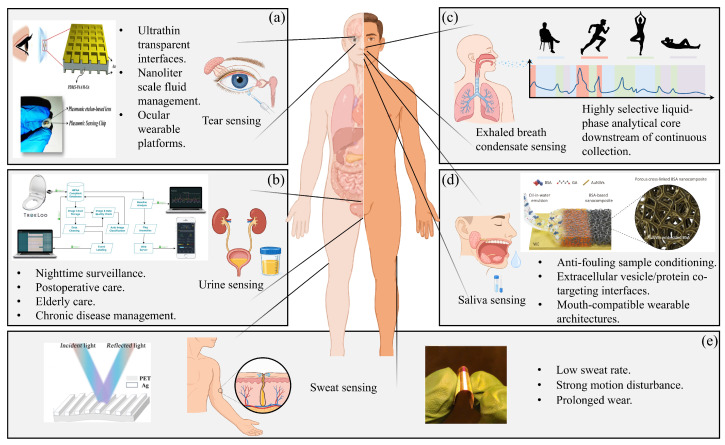
Future prospects of five body fluid fiber-optic SPR monitoring technology: (**a**) tear; (**b**) urine; (**c**) exhaled breath condensate; (**d**) saliva; (**e**) sweat. The pictures within each body fluid area respectively as follows: (**a**) real images of a simple lens based on PDMS-PAAM-SA polymer composite, and a plasmonic lens based on etalon nanostructure for glucose sensing [134]; (**b**) TrueLoo software block diagram; data captured by TrueLoo is uploaded to HIPAA-compliant databases and data flow begins. HIPAA: Health Insurance Portability and Accountability Act [135]; (**c**) full-day cross-activity in situ exhaled breath condensate (EBC) analysis of a healthy participant with EBC analysis and respiratory evaluation (EBCare) monitoring; (**d**) fabrication of emulsion-processed porous antifouling nanocomposite via nozzle-assisted printing. The AuNWs are embedded to the nanocomposite when BSA is cross-linked by glutaraldehyde (GA) [136]; (**e**) schematic diagram of the proposed flexible SPR sensor structure and photo of the flexible sensor prepared [137]. (Reprinted with permission, copyright of Springer Nature, Elsevier, and American Chemical Society).

From the current state of the field, rather than limited by whether high sensitivity can be demonstrated under ideal laboratory conditions, the central question of body fluid sensing has become how such sensitivity can be converted into high-confidence, high-stability, and clinically interpretable longitudinal readout in realistic settings. Accordingly, the most meaningful future directions will be determined by the interplay among fluid specific clinical targets, the nanomaterials best suited to the physicochemical constraints of each fluid, and the system architectures most likely to move from proof-of-concept devices to long-term wearable or home deployable platforms. As shown in Figure 10, the article will conduct a discussion of five aspects and look forward to their future development.

- **Tears**
  The future of tear sensing will depend heavily on the co-design of ultrathin transparent interfaces, nanoliter scale fluid management, and ocular wearable platforms rather than on pursuing a higher bulk RI sensitivity in isolation [138]. The ocular surface imposes strict requirements on oxygen permeability, visual safety, and mechanical softness. Recent developments in contact lenses, periocular patches, and eyewear-based sensors suggest a path toward continuous optical monitoring of glucose, lactate, pH, osmolarity, electrolytes, and, eventually, inflammatory proteins and vesicular biomarkers. As Roostaei et al. prepared plasma smart contact lenses on PDSM and polyacrylamide (PAAM) through soft nanolithography technology to complete the detection of glucose solutions with different concentrations [134] (Figure 10a). Beyond glucose, Eriş et al. developed an AuNP-decorated, lysozyme-imprinted SPR chip that reached an LOD of 0.008 μg/mL in artificial tear matrices, achieved approximately 97% recovery in complex samples, and retained performance over four regeneration cycles [139].
  Accordingly, transparent hydrogel matrices, PDMS/PAAM based contact-lens materials, ultrathin plasmonic nanocavity or fiber-end-facet structures, and graphene/${\mathrm{MoS}}_{2}$ type recognition layers are more promising than bulky fiber probes. Fiber SPR may be most useful when reconfigured into periocular miniaturized end-face probes, or hybrid systems that can be integrated with contact-lens-like carriers without compromising oxygen permeability, comfort, or vision quality [140]. In these platforms, high-Q plasmonic nanostructures may provide narrow-linewidth readout, while hydrogel or zwitterionic interfaces maintain hydration and suppress nonspecific adsorption.
- **Urine**
  Urine monitoring is suited for quasi-continuous, information-rich home monitoring in settings such as nighttime surveillance, postoperative care, elderly care, and chronic disease management. Compared with tears, urine offers higher information density but also harsher ionic and osmotic backgrounds that can distort SPR baselines and accelerate fouling. This makes deep, stable resonance dips and robust fitting behavior particularly important [141]. The most clinically valuable urinary targets may not be highly abundant but weakly specific small molecules; instead, extracellular vesicle subpopulations, disease associated protein fragments, and multi-metabolite signatures may offer far greater translation value [142]. Therefore, urine-oriented fiber SPR platforms should preferentially use salt-tolerant soft interfaces, Nafion or zwitterionic antifouling layers, MOF/GO or hydrogel MIP enrichment layers, and aptamer or antibody functionalized nanostructures for extracellular vesicle and protein fragment capture.
  As shown in Figure 10b, future fiber SPR systems for urine are therefore likely to be integrated with toilets, pads, catheters, or bedside collection modules, and to rely heavily on nanostructured enrichment layers and multimodal cross validation [135,143]. Its development should move beyond isolated small-molecule detection toward multi-marker panels combining extracellular vesicles, renal injury proteins, infection markers, and metabolic signatures, with automated sampling and cloud-based longitudinal interpretation. In addition, urine sensing can tolerate larger device volume and more complex fluid pretreatment, making it particularly suitable for high-Q multichannel SPR and reference-compensated fiber probes.
- **Exhaled Breath Condensate**
  EBC differs from the other body fluids in that its value lies in reflecting both airway status and systemic metabolism, but it is constrained by intrinsically low sample yield, extreme dilution, oral contamination, and historically poor access to onsite analysis [144]. Therefore, the most suitable SPR-related materials are not conventional thick biofunctional coatings, but high-Q plasmonic nanocavities, nanodisk/nanohole arrays, MOF-based enrichment layers, hydrogel-assisted condensation interfaces, and MIP or aptamer nanolayers capable of preconcentrating dilute targets.
  At the sensing-interface level, a 2026 numerical study proposed a BP-assisted SiC SPR architecture for acetone analysis in exhaled breath and EBC and used machine learning calibration to address nonlinear response [145]. Experimental progress toward a fiber-compatible platform was reported with an Ag/SnO_2_/GO coated SPR probe for exhaled acetone, which achieved a sensitivity of 0.83 nm/ppm, an 800 ppb detection limit, and response times of 12 s [146]. At the sampling level, patterned AuNP superlattice arrays integrated into a portable mask controlled evaporation-driven analyte localization and produced spatially uniform plasmonic EBC readout [147]. Although the latter platform uses surface-enhanced Raman scattering (SERS) rather than propagating SPR, its evaporation-control strategy is directly relevant to upstream enrichment and mass transport before fiber-SPR interrogation. Recently, Heng et al. developed EBCare, a mask-integrated platform that combines tandem passive cooling, automated microfluidics, highly selective electrochemical biosensors, and wireless readout circuitry to enable real-time, in situ and continuous multimodal monitoring of exhaled breath condensate biomarker, with demonstrated utility for assessing metabolic status and respiratory airway inflammation [8] (Figure 10c). In this scenario, fiber-optic SPR is more likely to function as a downstream liquid-phase analytical core integrated with mask-scale condensation and microfluidic refresh loops, rather than as a stand-alone front-end collector. Future exhaled breath condensate platforms should combine passive cooling or hydrogel-assisted collection, anti-evaporation fluid handling, narrow-linewidth optical readout, and selective enrichment materials to improve the reliability of airway inflammation, metabolic, and renal-function monitoring.
- **Saliva**
  For saliva, the decisive challenge will be the realization of stable preprocessing and disease-specific recognition under high viscosity, significant contamination, and strong inter-individual variability [148]. Saliva is easy to sample repeatedly and can contain proteins, RNA, extracellular vesicles (EV), and a range of small molecules, yet it is heavily affected by food residues, oral microbiota, mucins, and circadian rhythms [149]. Compared with purely sensitivity driven designs, porous albumin/AuNWs type antifouling nanocomposites and GO or MXene-assisted receptor interfaces are more relevant because they can simultaneously maintain conductivity, suppress nonspecific adsorption, and support biomarker capture.
  As a result, future fiber-optic SPR platforms for saliva should prioritize antifouling sample conditioning layers, EV/protein co-targeting interfaces, and mouth-compatible wearable or quasi-wearable architectures. Currently, the team led by Li et al. has created a porous nanocomposite sensor with a 1-micrometer-thick coating composed of cross-linked albumin and AuNWs through nozzle printing of oil-in-water emulsions, and has achieved long-term, anti-pollution monitoring of multiple secretions [136] (Figure 10d). Meanwhile, functionalized arrays of spherical and anisotropic AuNPs have been reported to exhibit shape and surface-chemistry-dependent LSPR responses, enabling discrimination among four oral bacterial species [150]. Clinical translation is also beginning to emerge. Annunziata et al. validated a three-arm POF-SPR prototype for salivary MIP-1α in 50 subjects, achieving an LOD of 0.15 pM and demonstrating a strong positive correlation with ELISA (enzyme-linked immunosorbent assay) measurements [151]. Rather than another single salivary protein sensor, the most meaningful direction may lie in multiplexed EV/protein panels for oral cancer, inflammatory diseases, and neurodegenerative conditions [152].
- **Sweat**
  In sweat sensing, the major competitive frontier will shift from demonstrating that a single analyte is measurable to achieving interpretable multi-analyte readout under low sweat rate, strong motion disturbance, and prolonged wear. Sweat is likely to remain the first body fluid to reach large scale continuous use because sampling is noninvasive and inherently compatible with skin mounted devices, while the analyte panel naturally spans electrolytes, glucose, lactate, urea, uric acid, and stress-related molecules [20,153,154]. Chen et al. further combined a dispersion-engineered hyperbolic metamaterial with a pH-responsive hydrogel on an optical fiber SPR probe, achieving pH sensitivities of −64.04 and −30.63 nm/pH across two pH intervals, selectivity against urea, NaCl, and glucose, and long-term storage stability [155]. The skin mounted format requires the sensing material to be flexible, adhesive, breathable, and mechanically robust. Therefore, PDMS, PET, silk, cellulose nanocomposite hydrogels, PVA, and other flexible substrates should be combined with plasmonic nanostructures such as NPs, NWs, NRs. In parallel, patterned AuNP superlattice films with domain sizes below the critical fracture size suppressed cracking and sustained uniform plasmonic signals during wearable sweat monitoring [156]. In this context, 2D/high-index multilayers and enzyme or boronic-acid functionalized interfaces are suitable for monitoring metabolite, sodium, chloride, and cortisol-related biomarkers.
  The main translational bottlenecks, however, are that sweat rate, evaporation, skin temperature, motion artifacts, and salinity fluctuations all distort the SPR baseline simultaneously. This makes narrow linewidth architecture and algorithmically assisted self-calibration more important than raw sensitivity alone. For instance, in 2025, a wearable flexible SPR sensor developed by Chai shows excellent robustness and mechanical stability under different bending radii and incident angles and has a narrow linewidth of about ∼6.9 nm and a sensitivity of 462.31 nm/RIU (Figure 10e). Meanwhile, the sensor integrates a microfluidic chip to achieve real-time monitoring of different analytes [137]. The most promising near-term solutions are therefore likely to be multiplexed sweat panels in which metabolites are interpreted together with internal calibration channels such as pH, temperature, conductivity, or sodium.

Ultimately, different body fluids will likely require distinct system morphologies. Sweat monitoring may favor skin-mounted multiplexed arrays; urine analysis may be better suited to quasi-continuous home or toilet-integrated platforms; saliva may require oral enrichment modules for extracellular vesicles, proteins, or pathogens; tear analysis will require transparent and mechanically compliant optical interfaces; and exhaled breath condensate may depend on mask integrated collection-analysis loops. These morphologies should be viewed as biofluid specific system templates, into which fiber-optic SPR modules can be integrated only when the optical architecture, sampling route, bio-interface chemistry, and clinical use case are mutually compatible. Accordingly, nanomaterial enhancement should be evaluated not merely by whether it increases sensitivity under idealized RI tests, but by whether it enables each biofluid to converge toward a reproducible, physiologically compatible, and clinically interpretable SPR sensing architecture.

## 5. Conclusions

Overall, nanomaterial-enabled functional enhancement is redefining the technological positioning of fiber-optic SPR in continuous body fluid monitoring. The goal is no longer limited to increasing peak shift or bulk RI sensitivity in a single assay. Instead, the field is moving toward a coordinated design framework in which mode enhancement, interface engineering, analyte enrichment/mass transport, and flexible integration work together to transform fiber SPR from a high sensitivity analytical method into a longitudinal readout platform for realistic human-centered scenarios. Traditional thin film SPR is inherently limited by penetration depth, linewidth, antifouling performance, and adaptation to complex biofluids; noble-metal nanostructures, two-dimensional materials, high index semiconductors, MOFs, and intelligent hydrogels are now addressing these weaknesses from complementary directions including local field amplification, receptor immobilization, molecular sieving, accelerated transport, and soft, conformal contact.

Future evaluation criteria will therefore move beyond bulk RI sensitivity toward peak trackability over extended operation, resistance to drift, inter-sample consistency, and clinical relevance. At the same time, meaningful translation of fiber-optic SPR into clinical and home health management will require continued progress in material optimization, interfacial recognition, system integration, manufacturing reproducibility, and longitudinal validation. The most successful nanomaterial strategies will be those that do not merely produce larger spectral shifts, but instead enable stable, interpretable, and deployable sensing performance in the real body fluid environments for which these technologies are ultimately intended. 

## Figures and Tables

**Table 1:** Common performance parameters used to evaluate fiber-optic SPR sensors.

Performance Parameters	Parametric Meaning
RI Sensitivity $\left(S_{xn}\right)=\frac{\partial x_{\mathrm{res}}}{\partial n}$	Shift of the resonance signal $\left(\Delta x_{\mathrm{res}}\right)$ induced by the change in the external RI (Δn).
Resolution	Smallest measurable change in the resonance signal can be reliably distinguished by the readout system.
FWHM	Full width at half maximum of the resonance dip. A narrower linewidth generally improves peak tracking and precision.
Figure of Merit $\left(FOM\right)=\frac{S_{xn}\times \left(1-R_{\min}\right)}{FWHM}$	Integrates sensitivity and spectral sharpness into one metric; $R_{\min}$ is the minimum normalized reflectance at resonance.
Detection Accuracy $\left(D.A.\right)=\frac{x_{\mathrm{res}}}{FWHM}$	Often taken as $\frac{1}{FWHM}$ or an equivalent linewidth-dependent term; reflects the ease of assigning the resonance position.
Quality Factor $\left(Q.F.\right)=\frac{\delta x_{\mathrm{res}}\times S_{xn}}{FWHM}$	A broader descriptor of resonance sharpness.
Signal-to-Noise Ratio $\left(SNR\right)=\frac{\partial x_{\mathrm{res}}}{FWHM}$	A signal quality evaluation index inversely proportional to FWHM.
Limit of Detection $\left(LOD\right)=\frac{\Delta \lambda }{S_{C_{0}}}$	Minimum detectable analyte concentration or RI change; Δλ is the wavelength resolution of the spectrometer, $S_{C_{0}}$ is the sensitivity at an analyte concentration near zero.
Limit of Quantification $\left(LOQ\right)=\frac{\delta x_{\mathrm{res}}}{S_{C_{0}}},\;\delta x_{\mathrm{res}}=i\sigma$	Lowest detectable analyte concentration; σ and *i* are the positive integer and the standard deviation in resonance signal near blank concentration, respectively.

**Table 2:** The sensing performance of some mode-enhancing fiber-optic SPR.

	Category	Key Performance Metrics	Application	Ref.
Metal film LSPR coupling	D-shaped fiber with Ag/α-Fe_2_O_3_ nanolayer	RI sensitivity (RI: 1.33–1.40): 8518 nm/RIU	Chemical solution testing	[97]
	Ag film/polydimethylsiloxane (PDMS) deposited on PCF	RI maximum sensitivity: 9000 nm/RIU; temperature maximum sensitivity: 7 nm/°C	Liquid dual-parameter sensing	[98]
	AuNPs/Au film	LOD: 50 CFU/mL; Linear range: 10^2^–10^8^ CFU/mL	Staphylococcus aureus	[99]
	AuNPs combined with PCF and Protein A	RI sensitivity: 3915 nm/RIU; LOD: 37 ng/mL	Human IgG	[22]
	Au film/AuNPs/Ti_3_C_2_Tx MXene	RI sensitivity: 2804.5 nm/RIU; FOM: 17.8279 ${\mathrm{RIU}}^{-1}$; IgG sensitivity: 1.7046 nm/(μg/mL)	Human IgG	[100]
	AuNPs/GO composite	Linear range: $10^{-10}$–$10^{-6}$ g/mL; LOD: 0.038 ng/mL	IgG	[60]
	Ag@Au/GO fiber	RI sensitivity: 4715.9 nm/RIU; IgG sensitivity: 0.53 nm/(μg/mL); LOD: 0.037 μg/mL	Human IgG	[61]
Anisotropic plasmonic and semiconductor nanostructures	Au film/AuNRs	Acetylcholine sensitivity (0.5–5 μg/mL): 0.4912 nm/(μg/mL); LOD: 0.45 μg/mL	Acetylcholine	[101]
	Multiple D-shaped fiber with AuNWs/Fe_3_O_4_	Resolution: $1.17\times 10^{-6}$ RIU	Biosensing	[77]
	Ag/ZnO NRs/GO_x_	Operating range: 0–10 mM; LOD: 0.012 mM	Dextrose/glucose-related diagnosis	[76]
	AuNRs with laser heterodyne feedback and frequency multiplexing compensation	RI resolution: $1.06\times 10^{-6}$ RIU	Fluoroquinolones	[79]
	Metallic ND-in-elliptical nanohole binary array	Sensitivity: 669 nm/RIU; FWHM: 5.8 nm; FOM: 115.3	Biosensing	[80]
	AuNWs with U-shaped open channel PCF	Maximum wavelength sensitivity: 7857.14 nm/RIU; amplitude sensitivity: −1893.08 ${\mathrm{RIU}}^{-1}$	MCF-7 breast cancer cells	[82]
	Dual channel D-shaped PCF with Au/${\mathrm{TiO}}_{2}$ bilayer and AuNPs array	Maximum RI sensitivity: 16,600 nm/RIU; LOD: $6.02\times 10^{-6}$ RIU; Q.F.: 71.9 ${\mathrm{RIU}}^{-1}$	Biosensing	[81]
	AuNSs with tip-enhanced local field	Minimum saturation intensity: 0.4 MW/${\mathrm{cm}}^{2}$; SA modulation depth: 53%	Compress the pulse width	[23]
2D/high-index multilayer	Graphene/${\mathrm{MoS}}_{2}$/ZnO multilayer	Maximum sensitivity: 4740.9 nm/RIU; FWHM: 81.69 nm; FOM: 51.71	Bioconcentration analysis	[87]
	InSe nanosheets	Sensitivity: 4655.76 nm/RIU; FOM: 33.25 ${\mathrm{RIU}}^{-1}$	Lower biomolecule concentration monitoring	[90]
	PtS_2_ overlayer with symmetrical U-tapered hollow-core fiber	RI sensitivity (RI: 1.33–1.37): 13,210 nm/RIU	Gastric carcinoma biomarker	[91]
	${\mathrm{MoS}}_{2}$-graphene heterostructure with pyrene-1-boronic acid	Sensitivity (0–500 mg/dL): 12,593.06 nm/RIU	Glucose	[92]
	${\mathrm{MoS}}_{2}$ nanosheets coating on bimetallic layers	RI sensitivity increases with the number of layers of MoS_2_; broadening and deformation of the spectrum	Solutions RI measuring	[94]
	MOF/GO composite	RI sensitivity: 4248.64 nm/RIU; LOD: 3.419 pM	${\mathrm{Pb}}^{2+}$	[93]
	U-shaped plastic optical fibers (POFs) with GO/polyvinyl alcohol (PVA)	RI sensitivity: 3949.14 nm/RIU; maximum detection sensitivity: 16.10 nm/(mg/mL); average response time: 3 min	Hemoglobin	[95]
	β-cyclodextrin and GO-enhanced SPR interface	RI sensitivity: 2910 nm/RIU; LOD: 2 nM; response time: 30s	Cholesterol	[96]

**Table 3:** Representative interfacial recognition-enhancing nanomaterials for fiber-optic SPR biosensing.

Material Class	Recognition-Enhancing Mechanism	Current Limitations
Graphene-family interfaces (e.g., graphene, GO, and rGO)	Large specific surface area, π-π interactions, oxygen-containing functional groups facilitate the immobilization of antibodies, aptamers, enzymes, and small-molecule receptors.	GO/rGO suffer from batch-dependent defect density and heterogeneous oxygen functional groups; swelling, aging, and long-term interfacial instability in aqueous media.
Reactive 2D materials (e.g., ${\mathrm{MoS}}_{2}$, ${\mathrm{WS}}_{2}$, ${\mathrm{Ti}}_{3}$$C_{2}$$T_{x}$, MXene, black phosphorus (BP), and ${\mathrm{PtS}}_{2}$)	Surface terminations, layered structures, strong molecular adsorption, and exciton–plasmon coupling improve interfacial capture and optical transduction.	MXenes/BP are susceptible to oxidation, hydration-induced degradation, and drift in real biofluids.
High-index and semiconducting recognition layers (e.g., ZnO, ${\mathrm{TiO}}_{2}$, ITO, and CuO)	High-RI semiconducting nanolayers enhance local field overlaps and provide stable scaffolds for enzymes, antibody, or aptamer immobilization. Some materials also contribute catalytic or selective adsorption functions.	Excessive layer thickness can introduce optical loss, broaden the resonance dip, and reduce peak traceability.
Permselective and adhesive polymer interfaces (e.g., polydopamine (PDA), Nafion, chitosan, and polypyrrole)	PDA provides universal adhesion and secondary reaction sites; Nafion offers ion-exchange selectivity and crosstalk suppression; imprinted or conductive polymers create selective recognition microenvironments.	Thick polymer layers may increase diffusion resistance and reduce coupling with the SPR near field.
Quantum dots (QDs) and functional nanodots (e.g., carbon QDs (CQDs), graphene QDs (GQDs), and metal-oxide QDs)	Abundant carboxyl, amino, hydroxyl, or other surface groups provide binding sites for ions, small molecules, or biological receptors.	Batch reproducibility, surface-group stability, and validation in real body fluids remain insufficient.
Molecularly imprinted polymer (MIP) and aptamer-based interfaces (e.g., MIP NPs, PBA, β-CD, and receptor proteins)	Artificial recognition cavities, aptamer conformation changes, boronate affinity, host–guest interactions, or receptor–ligand binding provide high target selectivity.	Aptamer stability, MIP binding-site accessibility, regeneration, and long-term antifouling performance in physiological fluids require further validation.
Stimuli-responsive soft interfaces (e.g., hydrogels, pH-responsive gels, enzyme-loaded hydrogels, MIP nanogels)	Swelling, ionization, enzymatic reaction, or target-induced deformation enables soft interfacial recognition.	Dehydration, swelling hysteresis, mechanical fatigue, and baseline drift remain major barriers.

**Table 4:** Representative enrichment, antifouling, and rapid mass-transport strategies for fiber-optic SPR biosensing.

Core Scheme	Key Performance Metrics	Application	Main Limitations	Ref.
Dual-channel Au-coated microstructured hollow fiber (internal/external channels).	RI range: 1.333–1.385; Sensitivity: 5645 nm/RIU (internal) and 1576 nm/RIU (external).	Microfluidic RI sensing.	Demonstrated with RI standards rather than target biofluids; the bare channels do not provide selective enrichment or antifouling.	[28]
Fe_3_O_4_@Au@PDA magnetic core-shell NP system.	LOD: 0.61 ng/mL; linear range: 1–200 ng/mL; SPR signal enhancement: ∼3.3-fold.	Tebuconazole detection and magnetic preconcentration.	Requires magnetic handling and an immunoassay workflow; long-term operation and antifouling in physiological fluids were not evaluated.	[116]
ZIF-8/lipase porous composite coating on a fiber SPR probe.	Sensitivity: 14.75 nm/mM; linear range: 0.1–3 mM; LOD: 0.096 mM; sample volume: 100 μL; stability variation: 0.47 nm.	Tributyrin detection.	Enzyme activity and MOF-film integrity may deteriorate during prolonged aqueous operation; wearable and biofluid validation is limited.	[121]
UiO-66-NH_2_-modified heterogeneous-core fiber SPR interface for DNA hybridization.	Range: 1 pM–1 μM; sensitivity: −7.72 nm/log(μM); LOD: 3 pM.	DNA detection.	Requires probe-DNA immobilization and hybridization; regeneration, fouling resistance, and long-term mechanical stability were not established.	[122]
AgNPs@Cu-TCPP(Pt)/Au/TFBG multichannel sensor with aptamer switching and MOF sieving.	Simultaneous detection within 6 min; LODs: Pb^2+^ 0.007 nM, Cd^2+^ 0.012 nM, and Hg^2+^ 0.005 nM.	Real-time multicomponent heavy-metal-ion detection.	Complex multilayer and multichannel fabrication; performance under long-term biofluid exposure and repeated wearable deformation remains unverified.	[123]
pH-responsive hydrogel on an MMF–SMF–MMF SPR probe.	pH range: 1–12; maximum sensitivity: 13 nm/pH at pH 8–10; weak temperature influence over 20–40 °C.	pH sensing.	Hydrogel dehydration, swelling hysteresis, mechanical fatigue, and calibration drift can limit long-term use.	[125]
Ag/Si SPR fiber coated with enzymes/coenzymes-entrapped hydrogel.	Range: 0–10 mM; maximum sensitivity: 21.70 nm/mM; resonance shift: 30 nm; LOD: 15.34 μM; LOQ: 29.63 μM.	Ethanol detection.	Enzyme/coenzyme aging and hydrogel dehydration may impair long-term calibration; continuous antifouling in body fluids was not tested.	[117]
Deformable transferrin-imprinted nanoMIPs on a plasmonic POF.	LOD: 1.2 fM; linear range: 1.2 fM–1.8 pM; Young’s modulus increased from 17 ± 6 to 56 ± 18 kPa after binding.	Human transferrin detection.	Hydration equilibration, regeneration, binding-site accessibility, and longitudinal antifouling require further validation.	[118]
Covalently immobilized α-casein nanoMIPs on an SPR chip.	LOD: 0.127 ppm (127 ± 97.6 ng/mL); range: 0–150 ppm; $K_{D}\approx 10$ nM; recovery: 87–120%.	α-Casein detection in cleaning-in-place wash samples.	Validated for food-process wash matrices rather than continuous physiological-fluid monitoring; long-term regeneration and fouling remain unresolved.	[119]

**Table 5:** Representative material and device strategies supporting flexible integration and continuous monitoring in fiber-optic SPR biosensing.

Core Scheme	Key Performance Metrics	Application	Main Limitations	Ref.
Uniaxially stretched metal/PCL plasmonic film with periodic nanogaps.	Average nanogap: ∼20 nm; SERS signal enhancement: >10-fold versus the unstretched film; good temperature stability.	Flexible, conformal SERS detection on nonplanar surfaces.	Irreversible stretching and SERS readout are not equivalent to continuous fiber-SPR operation; repeated strain and biofluid aging were not assessed.	[127]
PDMS-coated SPR microcavity mounted on a fiber end facet.	Sensitivity: −0.20 nm/°C over 30–47 °C; precision: ∼0.02 °C; repeatability: ±0.3 °C over 105 min; response time: <2 s.	Miniature temperature sensing and wearable thermal monitoring.	Targets temperature rather than a biomarker; biofluid cross-sensitivity and long-term skin-contact stability were not evaluated.	[128]
HMM-SPR sensor integrated with an on-fiber graphene/PMMA photodetector.	Urea/glucose LODs: 0.372/0.019 (mg/mL); urea/glucose sensitivities: 0.242/1.059 nm/(mg/mL).	Direct electrical readout of urea and glucose in artificial sweat.	Selectivity, full-system power/data integration, calibration drift, and prolonged real-sweat operation remain insufficiently validated.	[129]
Capillary all-fiber SPR microfluidic chip with an inner-wall sensing zone.	Probe diameter: 125 μm; sensitivity: 0.205 nm/(μg/mL); LOD: 0.49 μg/mL.	Arctigenin detection and compact flexible integration.	Analyte-specific proof of concept; antifouling, mechanical cycling, and continuous biofluid stability were not reported.	[30]
Carbonized AuNP-silk conductive patch for dual electrochemical/LDI-MS analysis.	Conductivity: (109.24 ± 13) × $10^{-3}$ S/cm; range: 0–100 mM; LOD: 20 mM (electrochemical) and 8 mM (LDI-MS); CKD cut-off: 60 mM.	Noninvasive sweat-urea detection.	Not a fiber-SPR device; relatively high LOD and limited evidence for continuous longitudinal calibration.	[32]
Cladding-etched multimode PMMA POF with Au/ITO overlayer.	RI range: 1.33–1.37; sensitivity: 2258 nm/RIU; FWHM: 219 nm; FOM: 10.13 $RIU^{-1}$; resolution: 2.74 × $10^{-4}$ RIU; maximum drift: 1.2 nm over 11 days.	Aqueous RI/biosensing.	Validated mainly in bulk-RI solutions; broad linewidth and wearable performance remain unresolved.	[132]
Three-channel PCF platform with Ag/MoS_2_ DNA probe, PAA/chitosan hydrogel pH probe, and PDMS temperature probe.	Selective cDNA sensitivity: 0.22 nm/(nmol/L); three sensing channels operated independently.	DNA hybridization with pH and temperature compensation.	Complex fabrication; long-term stability and real-biofluid validation were not reported.	[133]

## Data Availability

No new data were created or analyzed in this review.

## References

[1] Suryaprabha T., Choi C., Wu Y. (2026). Smart wearable and implantable biosensors for continuous health monitoring: Materials, biocompatibility, and AI integration. npj Flex. Electron..

[2] Jha R., Mishra P., Kumar S. (2024). Advancements in optical fiber-based wearable sensors for smart health monitoring. Biosens. Bioelectron..

[3] Hsueh W.T., Yu C.X., Cheng H.C. (2025). A comprehensive review of wearable devices for non-invasive biosensing. TrAC Trends Anal. Chem..

[4] Yang D.S., Ghaffari R., Rogers J.A. (2023). Sweat as a diagnostic biofluid. Science.

[5] Simard M., Nshimiyimana R., Chiang N. (2024). A potent proresolving mediator 17R-resolvin D2 from human macrophages, monocytes, and saliva. Sci. Adv..

[6] Jarnda K.V., Wang D., Qurrat-Ul-Ain (2023). Recent advances in electrochemical non-enzymatic glucose sensor for the detection of glucose in tears and saliva: A Review. Sens. Actuators A Phys..

[7] Wu Z., Liu R., Chen J. (2025). An enzymatic cleavage-triggered minimally invasive nanosensor for urine-based detection of early atherosclerosis. Sci. Adv..

[8] Heng W., Yin S., Min J. (2024). A smart mask for exhaled breath condensate harvesting and analysis. Science.

[9] Chen S., Guo Z., Lu B. (2025). A wearable device for continuous immunoassay-based monitoring of C-peptide in interstitial fluid. Sci. Adv..

[10] Singh S.U., Chatterjee S., Lone S.A. (2022). Advanced wearable biosensors for the detection of body fluids and exhaled breath by graphene. Microchim. Acta.

[11] Johnston L., Wang G., Hu K. (2021). Advances in Biosensors for Continuous Glucose Monitoring Towards Wearables. Front. Bioeng. Biotechnol..

[12] Min J., Tu J., Xu C. (2023). Skin-Interfaced Wearable Sweat Sensors for Precision Medicine. Chem. Rev..

[13] Hojjat Jodaylami M., Masson J.F., Badia A. (2025). Surface plasmon resonance sensing. Nat. Rev. Methods Prim..

[14] Halas N.J., Lal S., Chang W.S. (2011). Plasmons in Strongly Coupled Metallic Nanostructures. Chem. Rev..

[15] Zhang H., Gao Z., Zhang Y. (2025). Research Progress on Nanomaterials in SPR Sensors. Nanomaterials.

[16] Bakhshpour M., Göktürk I., Bereli N. (2021). Selective Detection of Penicillin G Antibiotic in Milk by Molecularly Imprinted Polymer-Based Plasmonic SPR Sensor. Biomimetics.

[17] Wu Q., Li N., Wang Y. (2019). A 2D transition metal carbide MXene-based SPR biosensor for ultrasensitive carcinoembryonic antigen detection. Biosens. Bioelectron..

[18] Guo X., Wang R., Liu F. (2024). Highly sensitive optical fiber SPR sensor based on chitosan for the detection of trace Cu^2+^ ion in aqueous solution. Opt. Quantum Electron..

[19] Yesudasu V., Pradhan H.S., Pandya R.J. (2021). Recent progress in surface plasmon resonance based sensors: A comprehensive review. Heliyon.

[20] Amjad A., Xian X. (2025). Optical sensors for transdermal biomarker detection: A review. Biosens. Bioelectron..

[21] Liu Y., Peng W. (2021). Fiber-Optic Surface Plasmon Resonance Sensors and Biochemical Applications: A Review. J. Light. Technol..

[22] Wang B.T., Wang Q. (2018). Sensitivity-Enhanced Optical Fiber Biosensor Based on Coupling Effect Between SPR and LSPR. IEEE Sens. J..

[23] Chen S., Niu R., Gao Y. (2024). Tunable nonlinear absorption of gold nanostars and application as a saturable absorber. Opt. Laser Technol..

[24] Arcadio F., Seggio M., Pitruzzella R. (2024). An Efficient Bio-Receptor Layer Combined with a Plasmonic Plastic Optical Fiber Probe for Cortisol Detection in Saliva. Biosensors.

[25] Zhou Y., Huang X., Xia B. (2025). An ultrasensitive optical fiber SPR sensor enhanced by functionalized carbon quantum dots for Fe^3+^ measurement. J. Alloy. Compd..

[26] Vikas, Weitz I.S., Nobili L.G. (2024). Fiber Optic SPR Sensor Modified with Copper Oxide Nanoparticles for Highly Sensitive and Selective Detection of Dopamine. IEEE Sens. J..

[27] Yang L., Liu C., Liu W. (2024). Advanced High-Performance Biochemical Sensor: Synchronous SPR Excitation with Parallel Multiple Single-Mode Fibers (SMFs). Plasmonics.

[28] Liu Z., Yang X., Zhang Y. (2018). Hollow fiber SPR sensor available for microfluidic chip. Sens. Actuators B Chem..

[29] Silva A.T., Figueiredo R., Azenha M. (2023). Imprinted Hydrogel Nanoparticles for Protein Biosensing: A Review. ACS Sens..

[30] Wei Y., Liu C., Zhang Y. (2023). All-Fiber SPR Microfluidic Chip for Arctigenin Detection. IEEE Sens. J..

[31] Mitu S.A., Ahmed K., Bui F.M. (2023). Au-TiO_2_-Coated Spectroscopy-Based Human Teeth Disorder Detection Sensor: Design and Quantitative Analysis. Micromachines.

[32] Promphet N., Phamonpon W., Karintrithip W. (2023). Carbonization of self-reduced AuNPs on silk as wearable skin patches for non-invasive sweat urea detection. Int. J. Biol. Macromol..

[33] Zayats A.V., Smolyaninov I.I., Maradudin A.A. (2005). Nano-optics of surface plasmon polaritons. Phys. Rep..

[34] Shalabney A., Abdulhalim I. (2011). Sensitivity-enhancement methods for surface plasmon sensors. Laser Photonics Rev..

[35] Khalid-Salako F., Kurt H., Yüce M. (2025). Surface Plasmon Resonance Aptasensors: Emerging Design and Deployment Landscape. Biosensors.

[36] Yu X., Yuan Y., Zhou J. (2026). Sensitivity enhancement of surface plasmon resonance biosensors based on versatile nanostructures: Principle, fabrication, and illustrative applications. Microsyst. Nanoeng..

[37] Peng Y., Zhao Y., Hu X.G. (2020). Optical fiber quantum biosensor based on surface plasmon polaritons for the label-free measurement of protein. Sens. Actuators B Chem..

[38] Wang L., Gu Y., Hu X. (2011). Long-range surface plasmon polariton modes with a large field localized in a nanoscale gap. Appl. Phys. B.

[39] Ma J., Liu K., Jiang J. (2019). Theoretical and Experimental Investigation of an All-Fiber Waveguide Coupled Surface Plasmon Resonance Sensor with Au–ZnO–Au Sandwich Structure. IEEE Access.

[40] Zhou X., Chen K., Li L. (2017). Angle modulated surface plasmon resonance spectrometer for refractive index sensing with enhanced detection resolution. Opt. Commun..

[41] Zeng Y., Zhou J., Wang X. (2019). Wavelength-scanning surface plasmon resonance microscopy: A novel tool for real time sensing of cell-substrate interactions. Biosens. Bioelectron..

[42] Mias S., Camon H. (2008). A review of active optical devices: I. Amplitude modulation. J. Micromech. Microeng..

[43] Kaňok R., Ciprian D., Hlubina P. (2020). Surface Plasmon Resonance-Based Sensing Utilizing Spatial Phase Modulation in an Imaging Interferometer. Sensors.

[44] Jiang L., Zeng S., Xu Z. (2017). Multifunctional Hyperbolic Nanogroove Metasurface for Submolecular Detection. Small.

[45] Yang K., Chen Y., Yan S. (2023). Nanostructured surface plasmon resonance sensors: Toward narrow linewidths. Heliyon.

[46] Zhou J., Wang Y., Zhang G.J. (2024). State-of-the-art strategies of surface plasmon resonance biosensors in clinical analysis: A comprehensive review. Coord. Chem. Rev..

[47] D’Agata R., Bellassai N., Jungbluth V. (2021). Recent Advances in Antifouling Materials for Surface Plasmon Resonance Biosensing in Clinical Diagnostics and Food Safety. Polymers.

[48] Mauriz E. (2020). Low-Fouling Substrates for Plasmonic Sensing of Circulating Biomarkers in Biological Fluids. Biosensors.

[49] Treebupachatsakul T., Boosamalee A., Chaithatwanitch K. (2022). Generalized figure of merit for plasmonic dip measurement-based surface plasmon resonance sensors. Biomed. Opt. Express.

[50] Qaiser N., Suryaprabha T., Vetrano F. (2026). Mechanics and bio-interface engineering in flexible biosensors for continuous health monitoring. npj Flex. Electron..

[51] Zhao J., Guo H., Li J. (2019). Body-Interfaced Chemical Sensors for Noninvasive Monitoring and Analysis of Biofluids. Trends Chem..

[52] Kim N.H., Choi M., Kim T.W. (2019). Sensitivity and Stability Enhancement of Surface Plasmon Resonance Biosensors based on a Large-Area Ag/MoS_2_ Substrate. Sensors.

[53] Jing J., Liu K., Jiang J. (2022). Performance improvement approaches for optical fiber SPR sensors and their sensing applications. Photonics Res..

[54] Shrivastav A.M., Satish L., Kushmaro A. (2021). Engineering the penetration depth of nearly guided wave surface plasmon resonance towards application in bacterial cells monitoring. Sens. Actuators B Chem..

[55] Wan H., Cui H., Liu H. (2025). Surface plasmon resonance biosensor chips: Fabrications and pharmaceutical applications. J. Pharm. Biomed. Anal..

[56] Wang G., Wang Y., Chen L. (2010). Nanomaterial-assisted aptamers for optical sensing. Biosens. Bioelectron..

[57] Refki S., Hayashi S., Rahmouni A. (2016). Anticrossing Behavior of Surface Plasmon Polariton Dispersions in Metal-Insulator-Metal Structures. Plasmonics.

[58] Yu X., Yuan Y., Xu J. (2019). Strong Coupling in Microcavity Structures: Principle, Design, and Practical Application. Laser Photonics Rev..

[59] Chu Y., Crozier K.B. (2009). Experimental study of the interaction between localized and propagating surface plasmons. Opt. Lett..

[60] Chen C.H., Chiang C.Y., Wu C.W. (2021). Integrated Graphene Oxide with Noble Metal Nanoparticles to Develop High-Sensitivity Fiber Optic Particle Plasmon Resonance (FOPPR) Biosensor for Biomolecules Determination. Nanomaterials.

[61] Xu Q., Yin H., Cui M. (2025). An Enhanced Bimetallic Optical Fiber SPR Biosensor Using Graphene Oxide for the Label-Free and Sensitive Detection of Human IgG. Sensors.

[62] MacDairmid A.R., Gallagher M.C., Banks J.T. (2003). Structure of Dithiothreitol Monolayers on Au(111). J. Phys. Chem. B.

[63] Drozd M., Pietrzak M.D., Malinowska E. (2018). SPRi-Based Biosensing Platforms for Detection of Specific DNA Sequences Using Thiolate and Dithiocarbamate Assemblies. Front. Chem..

[64] Shahriari S., Sastry M., Panjikar S. (2021). Graphene and Graphene Oxide as a Support for Biomolecules in the Development of Biosensors. Nanotechnol. Sci. Appl..

[65] Lv J., Wang J., Yang L. (2024). Recent advances of optical fiber biosensors based on surface plasmon resonance: Sensing principles, structures, and prospects. Sens. Diagn..

[66] Isho B., Abe K.T., Zuo M. (2020). Persistence of serum and saliva antibody responses to SARS-CoV-2 spike antigens in COVID-19 patients. Sci. Immunol..

[67] Bachhuber F., Huss A., Senel M. (2021). Diagnostic biomarkers in tear fluid: From sampling to preanalytical processing. Sci. Rep..

[68] Meng C., Chen J., Sun X. (2023). Urine Immunoglobin G Greater Than 2.45 mg/L Has a Correlation with the Onset and Progression of Diabetic Kidney Disease: A Retrospective Cohort Study. J. Pers. Med..

[69] Grésillon S., Aigouy L., Boccara A.C. (1999). Experimental Observation of Localized Optical Excitations in Random Metal-Dielectric Films. Phys. Rev. Lett..

[70] Wu Q.L., Zhao Y., Zhang Y.N. (2019). High sensitive applied load measurement using optical fiber tapered-loop probe with SPR effect. Opt. Laser Technol..

[71] Mao P., Luo Y., Chen X. (2014). Design and optimization of multimode fiber sensor based on surface plasmon resonance. Numerical Simulation of Optoelectronic Devices, 2014.

[72] Kanso M., Cuenot S., Louarn G. (2008). Sensitivity of Optical Fiber Sensor Based on Surface Plasmon Resonance: Modeling and Experiments. Plasmonics.

[73] He X., Yi H., Long J. (2016). Plasmonic Crystal Cavity on Single-Mode Optical Fiber End Facet for Label-Free Biosensing. Appl. Phys. Lett..

[74] Wang H., Zhang B., Zhao Y. (2019). Integrated Effects of Near-Field Enhancement-Induced Excitation and Surface Plasmon-Coupled Emission of Elongated Gold Nanocrystals on Fluorescence Enhancement and the Applications in PLEDs. ACS Appl. Electron. Mater..

[75] Yang P., Portalès H., Pileni M.P. (2010). Ability to discern the splitting between longitudinal and transverse plasmon resonances in Au compared to Ag nanoparticles in close-packed planar arrays. Phys. Rev. B.

[76] Usha S.P., Shrivastav A.M., Gupta B.D. (2016). FO-SPR based dextrose sensor using Ag/ZnO nanorods/GOx for insulinoma detection. Biosens. Bioelectron..

[77] Kadhim R.A., Nawar A.H., Wu J. (2021). Plasmonic Refractive Index Sensor Based on a Multiple D-shaped Au/Fe_3_O_4_ Nanowire. 2021 IEEE Sensors.

[78] Chauhan M., Singh V.K. (2022). ZnO nanostructures coated no-core fiber refractive index sensor. Mater. Sci. Semicond. Process..

[79] Dai Z., Tan J., Zhou K. (2023). Optical fiber SPR biosensor with frequency multiplexing compensated laser heterodyne feedback for ultrasensitive detection of fluoroquinolones. Sens. Actuators B Chem..

[80] Chen Z., Huang X., Zhang H. (2024). Metallic Nanodisk-in-Elliptical Nanohole Array Based Fiber Tip for Enhanced Plasmonic Refractometric Sensing. Plasmonics.

[81] Hua F., Shi H., Chen Q. (2026). Gold Nanoparticle-Enhanced Dual-Channel Fiber-Optic Plasmonic Resonance Sensor. Sensors.

[82] Khodatars Dashtmian M.R., Fallahi V., Seifouri M. (2025). Gold Nanowire-Enhanced SPR-PCF Biosensor for High-Throughput Cancer Cell Detection in Near-Infrared. Plasmonics.

[83] Rozman N., Fan K., Padilla W.J. (2024). Symmetry-Broken High- Q Terahertz Quasi-Bound States in the Continuum. ACS Photonics.

[84] Yin Z., Jing X., Li K. (2024). Cascaded dual-channel broadband SPR fiber optic sensor based on Ag/ZnO and Ag/TiO_2_/PDMS films structure. Opt. Laser Technol..

[85] Liu X., Kang J.-H., Yuan H. (2018). Tuning of Plasmons in Transparent Conductive Oxides by Carrier Accumulation. ACS Photonics.

[86] Li K., Li S., Yin Z. (2024). Experimental study of SPR sensor performance enhancement by metal oxides. Infrared Phys. Technol..

[87] Carvalho I., Xavier R., Fim F. (2023). A Field-Enhancement Optical Fiber SPR Sensor Using Graphene, Molybdenum Disulfide, and Zinc Oxide. Plasmonics.

[88] Vahed H., Nadri C. (2019). Sensitivity enhancement of SPR optical biosensor based on Graphene–MoS_2_ structure with nanocomposite layer. Opt. Mater..

[89] Huang Y., Guo J., Kang Y. (2015). Two dimensional atomically thin MoS_2_ nanosheets and their sensing applications. Nanoscale.

[90] Yin B., Wang Q., Chen L.A. (2023). Highly sensitive fiber SPR sensor based on InSe nanosheets. Opt. Fiber Technol..

[91] Li B., Wang Q. (2024). Surface electric field enhanced biosensor based on symmetrical U-tapered HCF structure for gastric carcinoma biomarker trace detection. Biosens. Bioelectron..

[92] Ma J., Wang R., Li D. (2025). MoS_2_-Graphene heterostructure enhanced fiber optic SPR sensor for highly sensitive glucose detection. Microchem. J..

[93] Li L., Huang Y., Wu S. (2025). Metal-organic frameworks/graphene oxide synergistic enhanced optical fiber SPR sensor for ultra-trace lead ions detection. Opt. Lasers Eng..

[94] Wang Q., Jiang X., Niu L.Y. (2020). Enhanced sensitivity of bimetallic optical fiber SPR sensor based on MoS_2_ nanosheets. Opt. Lasers Eng..

[95] Al Noman A., Cheng X., O’Keeffe S. (2025). Graphene oxide modified U-shaped polymer optical fiber biosensor for hemoglobin sensing. Anal. Chim. Acta.

[96] Li X., Zhang J., Yan D. (2026). Highly Sensitive Cholesterol Detection Using *β*-Cyclodextrin and Graphene Oxide-Enhanced Surface Plasmon Resonance Sensor. IEEE Trans. Instrum. Meas..

[97] Mumtaz F., Roman M., Zhang B. (2022). A Simple Optical Fiber SPR Sensor with Ultra-High Sensitivity for Dual-Parameter Measurement. IEEE Photonics J..

[98] Li J., Jing X., Yin Z. (2025). Low-Crosstalk Refractive Index-Temperature Synchronized Detection Sensor Based on PCF-SPR in a Wide Temperature Domain. Plasmonics.

[99] Zhao C., Zhu J., Ma X. (2025). High sensitive optical fiber SPR biosensor enhanced by Ti_3_C_2_T_x_ MXene for label-free detecting Staphylococcus aureus. Microchem. J..

[100] Zhu J., Zhao C., Xia B. (2024). An Enhanced SPR Optical Fiber Biosensor Using Ti_3_C_2_T_x_ MXene/AuNPs for Label-Free and Sensitive Detection of Human IgG. Nanoscale.

[101] Zhang Y., Ding L., Wang S. (2024). A New Acetylcholine Optical Fiber Biosensor Based on Gold Film-GNRs Resonance Coupling Enhancement. IEEE Sens. J..

[102] Jalal N.R., Madrakian T., Ahmadi M. (2024). Wireless wearable potentiometric sensor for simultaneous determination of pH, sodium and potassium in human sweat. Sci. Rep..

[103] Timpel J., Klinghammer S., Riemenschneider L. (2023). Sensors for in situ monitoring of oral and dental health parameters in saliva. Clin. Oral Investig..

[104] Kaewpradub K., Veenuttranon K., Jantapaso H. (2025). A Fully-Printed Wearable Bandage-Based Electrochemical Sensor with pH Correction for Wound Infection Monitoring. Nano-Micro Lett..

[105] Brasier N., Wang J., Gao W. (2024). Applied body-fluid analysis by wearable devices. Nature.

[106] Singh S., Kumar R., Chaudhary B., Kumar D., Kumar S. (2026). Graphene Oxide-Based Surface Plasmon Resonance Sensor for Sensitivity Enhancement. VLSI, Microwave and Wireless Technologies;Lecture Notes in Electrical Engineering.

[107] Guo S., Garaj S., Bianco A. (2022). Controlling covalent chemistry on graphene oxide. Nat. Rev. Phys..

[108] Mathioudakis G.N., Visvini G.A., Sygellou L. (2025). Comparative In-Depth Investigation of Benchmark Graphene Oxides in the Perspective of Their Integration into Industrial Production Processes. Nanomaterials.

[109] Otsuka H., Urita K., Honma N. (2024). Transient chemical and structural changes in graphene oxide during ripening. Nat. Commun..

[110] Ma J., Ping D., Dong X. (2017). Recent Developments of Graphene Oxide-Based Membranes: A Review. Membranes.

[111] Xu X., Zhang Y., Wang B. (2018). A novel surface plasmon resonance sensor based on a functionalized graphene oxide/molecular-imprinted polymer composite for chiral recognition of L-tryptophan. RSC Adv..

[112] Cho S., Lee Y., Koh H. (2016). Superior Chemical Sensing Performance of Black Phosphorus: Comparison with MoS_2_ and Graphene. Adv. Mater..

[113] Cheng L., Guo R., Zheng W. (2025). ZnO-Nafion assisted optical fiber dual-SPR biosensor for simultaneous detection of urea and uric acid concentrations. Biosens. Bioelectron..

[114] Malic L., Sandros M.G., Tabrizian M. (2011). Designed Biointerface Using Near-Infrared Quantum Dots for Ultrasensitive Surface Plasmon Resonance Imaging Biosensors. Anal. Chem..

[115] Sen B., Sarma H. (2026). Carbon quantum dots as multifunctional nanomaterials for sustainable optoelectronic biosensing and green photonics. Commun. Mater..

[116] Zhang B., Li Y.L., Wang L. (2025). Magnetic Fe_3_O_4_@Au@PDA core-shell nanoparticle-enhanced SPR detection of tebuconazole. Microchem. J..

[117] Semwal V., Shrivastav A.M., Verma R. (2016). Surface plasmon resonance based fiber optic ethanol sensor using layers of silver/silicon/hydrogel entrapped with ADH/NAD. Sens. Actuators B Chem..

[118] Cennamo N., Maniglio D., Tatti R. (2020). Deformable molecularly imprinted nanogels permit sensitivity-gain in plasmonic sensing. Biosens. Bioelectron..

[119] Ashley J., Shukor Y., D’Aurelio R. (2018). Synthesis of Molecularly Imprinted Polymer Nanoparticles for *α*-Casein Detection Using Surface Plasmon Resonance as a Milk Allergen Sensor. ACS Sens..

[120] Shu Y., Ye Q., Dai T. (2021). Encapsulation of Luminescent Guests to Construct Luminescent Metal–Organic Frameworks for Chemical Sensing. ACS Sens..

[121] Zhang H., Li X., Zhou X. (2024). Sensitive and Label-Free Optical Fiber Biosensor Based on ZIF-8/Lipase Composite Material and Its Application in Tributyrin Detection. IEEE Trans. Instrum. Meas..

[122] Li L., Yu S., Jing C. (2024). Metal–Organic Frameworks Modified Optical Fiber SPR Biosensor for DNA Detection. IEEE Sens. J..

[123] Yan J., Liu R., Shi J. (2025). Ultra-sensitive, multi-component, and real-time detection of heavy metal ions via AgNPs@Cu-TCPP(Pt) sensitized strong coupling plasmonic sensor. Chem. Eng. J..

[124] Trabbic-Carlson K., Setton L.A., Chilkoti A. (2003). Swelling and Mechanical Behaviors of Chemically Cross-Linked Hydrogels of Elastin-like Polypeptides. Biomacromolecules.

[125] Zhao Y., Lei M., Liu S.X. (2018). Smart hydrogel-based optical fiber SPR sensor for pH measurements. Sens. Actuators B Chem..

[126] Liu C., Xu T., Wang D. (2020). The role of sampling in wearable sweat sensors. Talanta.

[127] Xu K., Wang Z., Tan C.F. (2017). Uniaxially Stretched Flexible Surface Plasmon Resonance Film for Versatile Surface Enhanced Raman Scattering Diagnostics. ACS Appl. Mater. Interfaces.

[128] Kong W., Sun X., Zhong H. (2025). Miniature fiber-optic temperature sensors using PDMS-coated surface plasmon resonance microcavities. Sens. Actuators A Phys..

[129] Shen C., Huang J., Hu S. (2025). Direct electronical readout of surface plasmon resonance biosensor enabled by on-fiber Graphene/PMMA photodetector. Biosens. Bioelectron..

[130] Pasquardini L., Cennamo N., Arcadio F. (2022). A Review of Apta-POF-Sensors: The Successful Coupling between Aptamers and Plastic Optical Fibers for Biosensing Applications. Appl. Sci..

[131] Chou Chao C.T., Chou Chau Y.F. (2026). Engineering plasmonic photonic crystal fiber sensors: Design, fabrication strategies, and performance roadmap. Mater. Sci. Eng. B.

[132] Woyessa G., Bang O. (2025). Sensitivity Enhancement of Polymer Optical Fiber Surface Plasmon Resonance Sensor Utilizing ITO Overlayer. Sensors.

[133] Yin Z., Zhang Z., Jing X. (2025). Near-infrared SPR biosensor based on photonic crystal fiber for DNA hybridization detection. Anal. Chim. Acta.

[134] Roostaei N., Hamidi S.M. (2025). Plasmonic smart contact lens based on etalon nanostructure for tear glucose sensing. Sci. Rep..

[135] Glenn J., Sarmadi P., Cristman P. (2024). Using the TrueLoo Smart Device to Record Toileting Sessions in Older Adults: Retrospective Validation and Acceptance Study. JMIR Aging.

[136] Lee J.C., Kim S.Y., Song J. (2024). Micrometer-thick and porous nanocomposite coating for electrochemical sensors with exceptional antifouling and electroconducting properties. Nat. Commun..

[137] Chai J., Lin Z., Wang Y. (2025). Low-Cost, High-Performance Refractive Index Sensing with Ultranarrow Resonance Line Width for Wearable Sensing. ACS Appl. Mater. Interfaces.

[138] Park W., Seo H., Kim J. (2024). In-depth correlation analysis between tear glucose and blood glucose using a wireless smart contact lens. Nat. Commun..

[139] Eriş Ş., Çimen D., Denizli A. (2025). Lysozyme-Imprinted Surface Plasmon Resonance Chips Decorated with Gold Nanoparticles for Lysozyme Detection. ACS Omega.

[140] Sun X., Lei Z., Zhong H. (2022). A quasi-3D Fano resonance cavity on optical fiber end-facet for high signal-to-noise ratio dip-and-read surface plasmon sensing. Light. Adv. Manuf..

[141] Liu L., Liu Z., Zhang Y. (2022). Multi-channel Optical Fiber Surface Plasmon Resonance Sensor with Narrow FWHM, High Figure of Merit, and Wide Detection Range. Plasmonics.

[142] Ding T., Li Y., Xue L. (2026). Identification and diagnostic evaluation of an aptamer targeting prostate-cancer-derived small extracellular vesicles. Mol. Ther. Nucleic Acids.

[143] Tasoglu S. (2022). Toilet-based continuous health monitoring using urine. Nat. Rev. Urol..

[144] Ibrahim W., Wilde M.J., Cordell R.L. (2022). Visualization of exhaled breath metabolites reveals distinct diagnostic signatures for acute cardiorespiratory breathlessness. Sci. Transl. Med..

[145] Juwel M.B., Utshob M.A.A.I., Bhuiyan M.T. (2026). ML-Assisted Accuracy Prediction of a Silicon Carbide-Based SPR Biosensor for Acetone Concentration Measurement. J. Am. Ceram. Soc..

[146] Prasanth A., Narasimman S., Velumani M. (2026). SnO_2_/GO coated SPR optical fiber sensor for acetone detection in diabetes management: Experimental and theoretical studies. Spectrochim. Acta Part A Mol. Biomol. Spectrosc..

[147] Ye H., Chen X., Ma L. (2026). Multimodal Evaporation on Patterned Nanoparticle Arrays for Exhaled Breath Condensate Detection. J. Am. Chem. Soc..

[148] Li Y., Ou Y., Fan K. (2024). Salivary diagnostics: Opportunities and challenges. Theranostics.

[149] Dongiovanni P., Meroni M., Casati S. (2023). Salivary biomarkers: Novel noninvasive tools to diagnose chronic inflammation. Int. J. Oral Sci..

[150] Wenck C., Leopoldt D., Habib M. (2024). Colorimetric detection of oral bacteria using functionalized gold nanoparticles as a plasmonic biosensor array. Nanoscale Adv..

[151] Annunziata M., Arcadio F., Stampone E. (2025). Plasmonic Optical Fibre-Based Point-of-Care Test for Periodontal MIP-1*α* Detection: A Validation Study of a Multiplexed Biosensor Prototype. Int. Dent. J..

[152] Lopez Baltazar J.M., Gu W., Yu Q. (2024). Enhancing Extracellular Vesicle Detection via Cotargeting Tetraspanin Biomarkers. Anal. Chem..

[153] Wang J., Luo Y., Zhou Z. (2024). Epidermal wearable optical sensors for sweat monitoring. Commun. Mater..

[154] Wang Y., Cheng S., Sun C. (2023). Organic Thin Film Transistor for Effective Biomarker Detection in Early Disease Diagnosis. Chemosensors.

[155] Chen Y., Hu S., Shen C. (2025). Hyperbolic-Metamaterial-Based Optical Fiber SPR Sensor Enhanced by a Smart Hydrogel for Perspiration pH Measurements. Nano Lett..

[156] Ye H., Chen X., Huang X. (2024). Patterned Gold Nanoparticle Superlattice Film for Wearable Sweat Sensors. Nano Lett..

